# Supplementary material for: A randomized control trial of high-dose micronutrient-antioxidant supplementation in healthy persons with untreated HIV infection
Source: PLoS One. 2022 Jul 14;17(7):e0270590. doi: 10.1371/journal.pone.0270590 (PMC9282469; doi:10.1371/journal.pone.0270590)
Supplement: S2 Fig — Week 0 includes all individuals who were screened in and allocated to a group. The table beneath shows the individuals remaining at risk at each time point and the number of individuals experiencing an event in that interval is in brackets. There were 29 events in the Control group (Black line) and 28 events in the Treatment group (Gray line) over the study period of 96 weeks. (PPTX) [file pone.0270590.s003.pptx]

## Slide 1
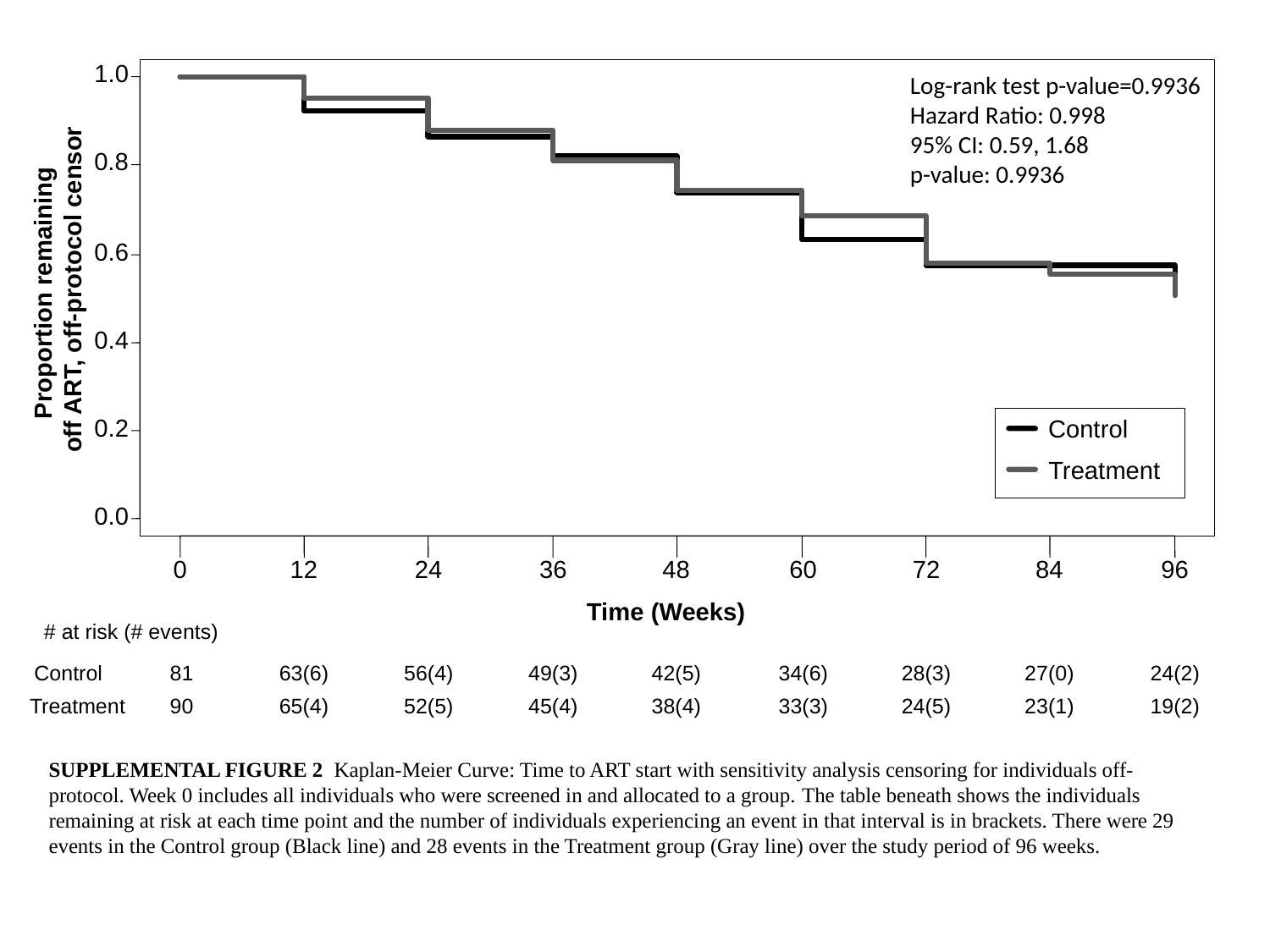

1.0
Log-rank test p-value=0.9936
Hazard Ratio: 0.998
95% CI: 0.59, 1.68
p-value: 0.9936
0.8
0.6
Proportion remaining
off ART, off-protocol censor
0.4
0.2
Control
Treatment
0.0
0
12
24
36
48
60
72
84
96
Time (Weeks)
# at risk (# events)
Control
81
63(6)
56(4)
49(3)
42(5)
34(6)
28(3)
27(0)
24(2)
Treatment
90
65(4)
52(5)
45(4)
38(4)
33(3)
24(5)
23(1)
19(2)
SUPPLEMENTAL FIGURE 2 Kaplan-Meier Curve: Time to ART start with sensitivity analysis censoring for individuals off-protocol. Week 0 includes all individuals who were screened in and allocated to a group. The table beneath shows the individuals remaining at risk at each time point and the number of individuals experiencing an event in that interval is in brackets. There were 29 events in the Control group (Black line) and 28 events in the Treatment group (Gray line) over the study period of 96 weeks.
