# Supplementary material for: A randomized control trial of high-dose micronutrient-antioxidant supplementation in healthy persons with untreated HIV infection
Source: PLoS One. 2022 Jul 14;17(7):e0270590. doi: 10.1371/journal.pone.0270590 (PMC9282469; doi:10.1371/journal.pone.0270590)
Supplement: S3 Fig — Week 0 includes all individuals who were screened in and allocated to a group. The table beneath shows the individuals remaining at risk at each time point and the number of individuals experiencing an event in that interval is in brackets. There were 32 events in the Control group (Black line) and 34 events in the Treatment group (Gray line) over the study period of 96 weeks. (PPTX) [file pone.0270590.s004.pptx]

## Slide 1
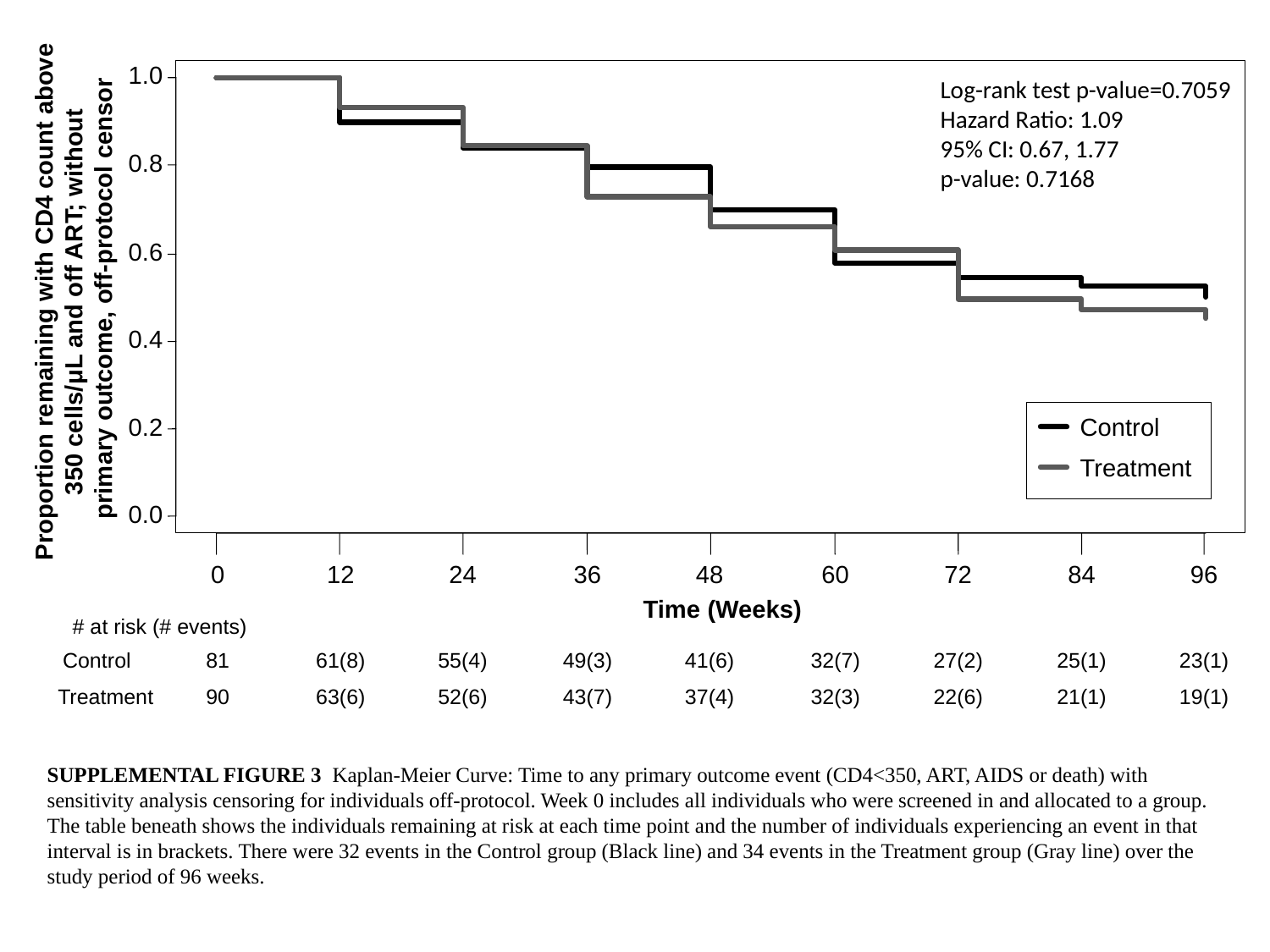

1.0
Log-rank test p-value=0.7059
Hazard Ratio: 1.09
95% CI: 0.67, 1.77
p-value: 0.7168
0.8
0.6
Proportion remaining with CD4 count above
350 cells/μL and off ART; without
primary outcome, off-protocol censor
0.4
0.2
Control
Treatment
0.0
0
12
24
36
48
60
72
84
96
Time (Weeks)
# at risk (# events)
Control
81
61(8)
55(4)
49(3)
41(6)
32(7)
27(2)
25(1)
23(1)
Treatment
90
63(6)
52(6)
43(7)
37(4)
32(3)
22(6)
21(1)
19(1)
SUPPLEMENTAL FIGURE 3 Kaplan-Meier Curve: Time to any primary outcome event (CD4<350, ART, AIDS or death) with sensitivity analysis censoring for individuals off-protocol. Week 0 includes all individuals who were screened in and allocated to a group. The table beneath shows the individuals remaining at risk at each time point and the number of individuals experiencing an event in that interval is in brackets. There were 32 events in the Control group (Black line) and 34 events in the Treatment group (Gray line) over the study period of 96 weeks.
