# Supplementary material for: A randomized control trial of high-dose micronutrient-antioxidant supplementation in healthy persons with untreated HIV infection
Source: PLoS One. 2022 Jul 14;17(7):e0270590. doi: 10.1371/journal.pone.0270590 (PMC9282469; doi:10.1371/journal.pone.0270590)
Supplement: S4 Fig — CD4 T lymphocyte counts of HIV-infected participants on 100% RDA (Control- A, C) versus high-dose (Treatment- B, D) supplements over time, confined to intent-to-treat analysis (data censored for those starting ART, ITT; A, B) or off-protocol censoring (OP; C, D). For graphs A-D, each line represents an individual participant’s measurements taken every 12 weeks until the end of the study (96 weeks or discontinuation of study) and only data from those participants with measurements for at least 36 weeks (i.e. having at least 3 data points) were included. Measurements at Week 0 represent the participant’s baseline CD4 T lymphocyte count prior to taking the indicated supplement. E) Mean change in CD4 T lymphocytes (cells/μL) over time (in Weeks) was calculated for Control and Treatment groups using linear mixed-effects model analysis. Data was censored for ITT or OP and the rate per 52 weeks is given. F) The mean difference in slope at 52 weeks for Control versus Treatment CD4 trajectories with the 95% confidence intervals are reported. Both ITT and OP censoring are reported with p-values. (PPTX) [file pone.0270590.s005.pptx]

## Slide 1
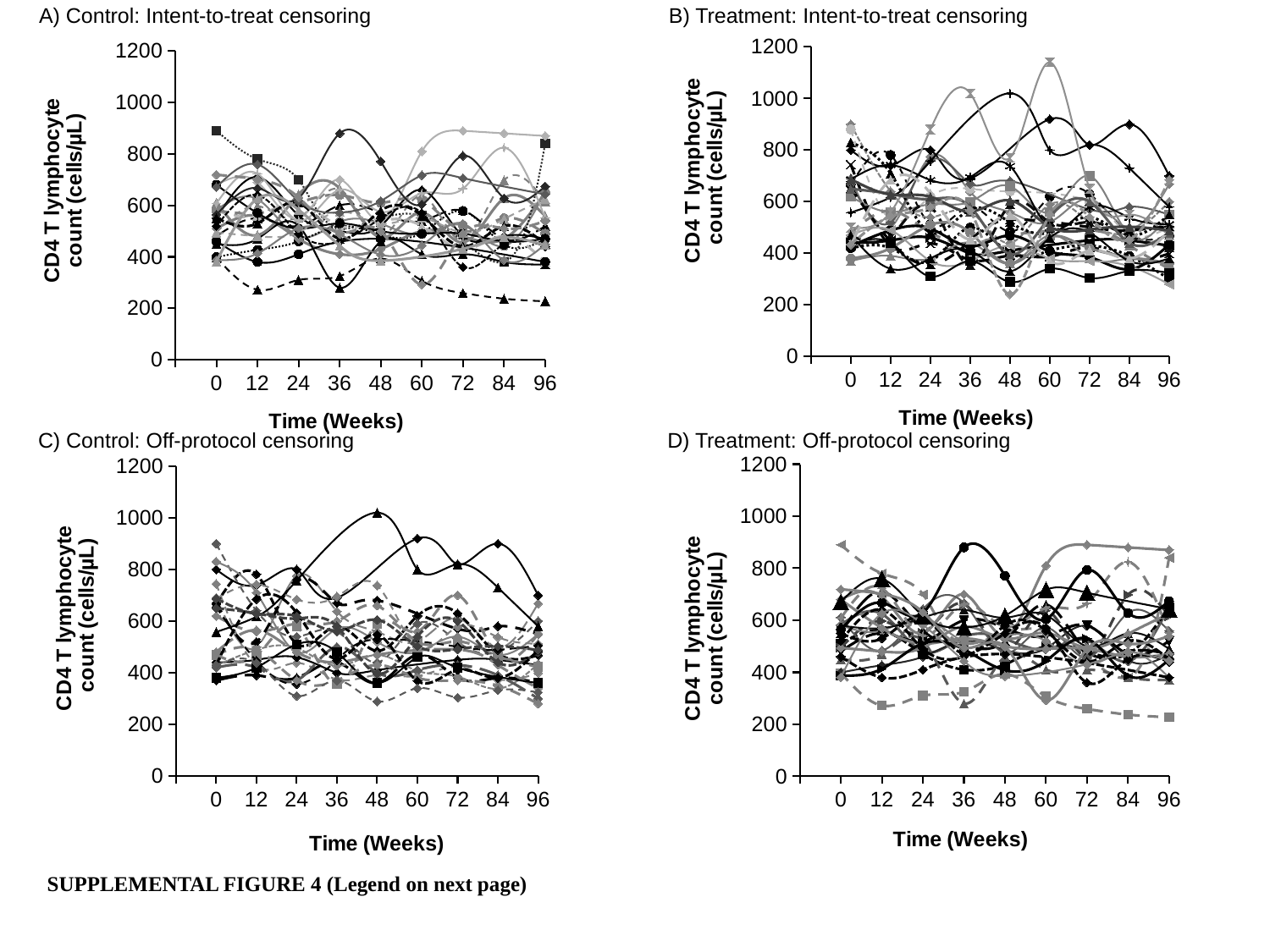

### Chart
| Category | 06-011 | 09-002 | 10-002 | 05-015 | 14-005 | 02-011 | 08-012 | 09-007 | 12-007 | 02-010 | 06-006 | 11-011 | 14-006 | 01-023 | 05-012 | 03-005 | 12-010 | 04-001 | 05-019 | 05-025 | 21-001 | 07-003 | 23-002 | 03-004 | 21-007 | 01-017 | 01-015 | 05-021 | 10-001 | 12-015 | 01-011 | 01-028 | 01-037 | 01-041 | 02-004 | 02-007 | 03-006 | 03-008 | 04-002 | 05-001 | 05-008 | 09-004 | 09-013 | 10-006 | 12-004 | 12-026 | 14-001 | 16-001 | 17-003 | 21-002 | 21-003 | 22-002 | 23-003 | 23-010 |
|---|---|---|---|---|---|---|---|---|---|---|---|---|---|---|---|---|---|---|---|---|---|---|---|---|---|---|---|---|---|---|---|---|---|---|---|---|---|---|---|---|---|---|---|---|---|---|---|---|---|---|---|---|---|---|A) Control: Intent-to-treat censoring
B) Treatment: Intent-to-treat censoring
### Chart
| Category | 03-003 | 07-006 | 01-014 | 01-030 | 01-031 | 05-017 | 11-002 | 12-023 | 11-010 | 01-024 | 02-008 | 04-003 | 05-020 | 07-002 | 01-013 | 01-026 | 05-018 | 03-007 | 10-011 | 01-025 | 01-039 | 02-009 | 05-023 | 11-009 | 23-007 | 01-034 | 14-003 | 23-006 | 01-001 | 01-003 | 01-009 | 01-016 | 02-001 | 02-005 | 02-006 | 03-001 | 05-009 | 05-022 | 08-002 | 09-005 | 09-006 | 10-003 | 11-005 | 11-006 | 12-001 | 12-003 | 12-005 | 12-011 | 12-017 | 14-004 | 21-004 | 21-008 | 22-001 | 23-004 | 23-009 |
|---|---|---|---|---|---|---|---|---|---|---|---|---|---|---|---|---|---|---|---|---|---|---|---|---|---|---|---|---|---|---|---|---|---|---|---|---|---|---|---|---|---|---|---|---|---|---|---|---|---|---|---|---|---|---|---|
C) Control: Off-protocol censoring
D) Treatment: Off-protocol censoring
### Chart
| Category | 01-030 | 05-017 | 11-002 | 12-023 | 11-010 | 01-026 | 01-024 | 02-008 | 04-003 | 05-020 | 07-002 | 10-011 | 01-039 | 02-009 | 05-023 | 01-001 | 02-001 | 11-009 | 01-013 | 03-007 | 01-025 | 05-009 | 23-007 | 01-034 | 14-003 | 23-006 | 01-003 | 01-009 | 01-016 | 02-005 | 02-006 | 03-001 | 05-022 | 08-002 | 09-005 | 09-006 | 10-003 | 11-005 | 11-006 | 12-001 | 12-003 | 12-005 | 12-011 | 14-004 | 21-004 | 21-008 | 22-001 | 23-004 | 23-009 |
|---|---|---|---|---|---|---|---|---|---|---|---|---|---|---|---|---|---|---|---|---|---|---|---|---|---|---|---|---|---|---|---|---|---|---|---|---|---|---|---|---|---|---|---|---|---|---|---|---|---|
### Chart
| Category | 06-011 | 09-002 | 10-002 | 05-015 | 14-005 | 02-011 | 08-012 | 09-007 | 12-007 | 02-010 | 06-006 | 11-011 | 14-006 | 01-023 | 05-012 | 03-005 | 12-010 | 04-001 | 05-019 | 05-025 | 21-001 | 07-003 | 23-002 | 03-004 | 21-007 | 01-017 | 01-015 | 05-021 | 10-001 | 12-015 | 01-011 | 01-028 | 01-037 | 01-041 | 02-004 | 02-007 | 03-006 | 03-008 | 04-002 | 05-001 | 05-008 | 09-004 | 09-013 | 10-006 | 12-004 | 12-026 | 14-001 | 16-001 | 17-003 | 21-002 | 21-003 | 22-002 | 23-003 | 23-010 |
|---|---|---|---|---|---|---|---|---|---|---|---|---|---|---|---|---|---|---|---|---|---|---|---|---|---|---|---|---|---|---|---|---|---|---|---|---|---|---|---|---|---|---|---|---|---|---|---|---|---|---|---|---|---|---|
SUPPLEMENTAL FIGURE 4 (Legend on next page)

## Slide 2
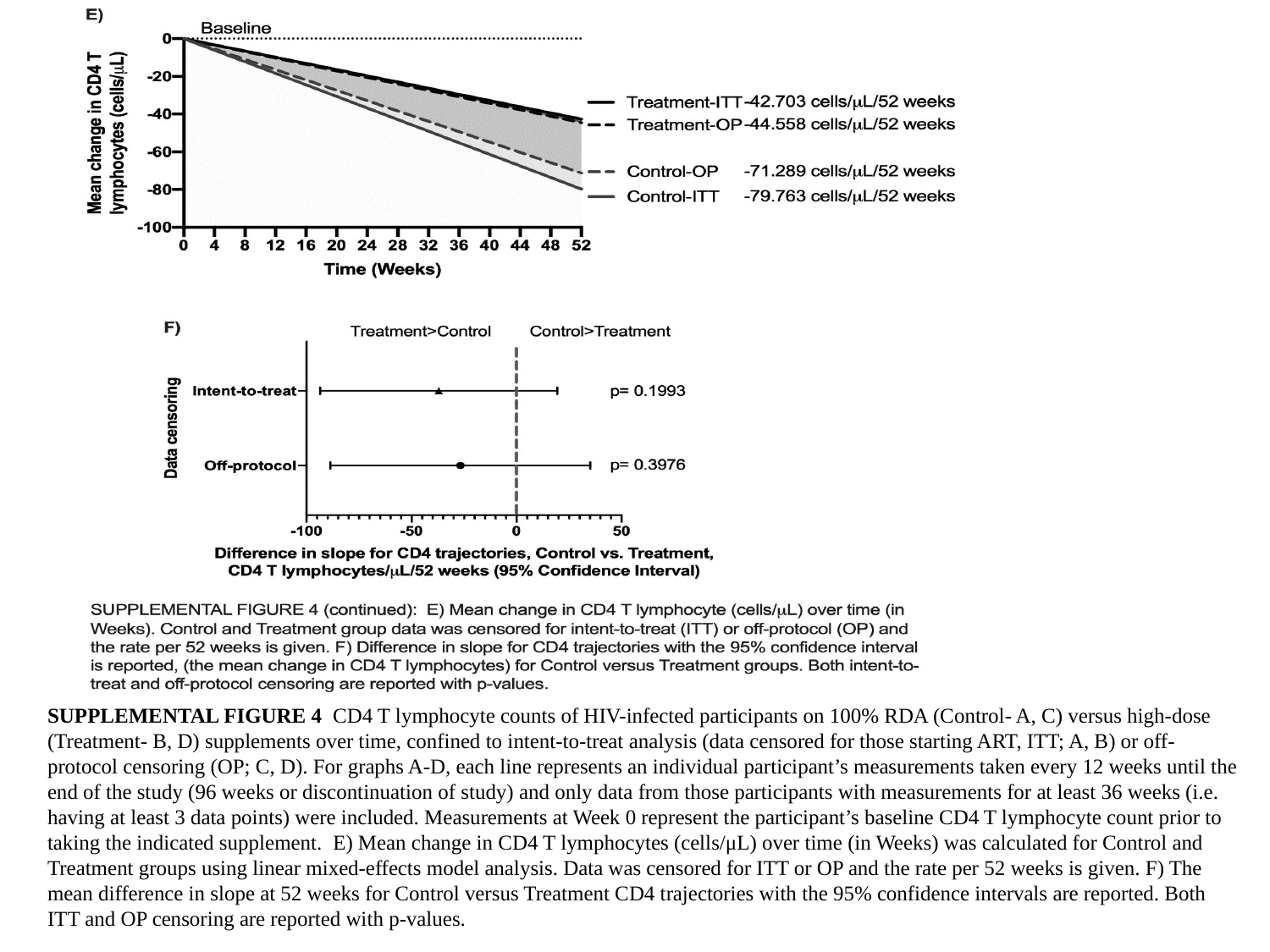

SUPPLEMENTAL FIGURE 4 CD4 T lymphocyte counts of HIV-infected participants on 100% RDA (Control- A, C) versus high-dose (Treatment- B, D) supplements over time, confined to intent-to-treat analysis (data censored for those starting ART, ITT; A, B) or off-protocol censoring (OP; C, D). For graphs A-D, each line represents an individual participant’s measurements taken every 12 weeks until the end of the study (96 weeks or discontinuation of study) and only data from those participants with measurements for at least 36 weeks (i.e. having at least 3 data points) were included. Measurements at Week 0 represent the participant’s baseline CD4 T lymphocyte count prior to taking the indicated supplement. E) Mean change in CD4 T lymphocytes (cells/μL) over time (in Weeks) was calculated for Control and Treatment groups using linear mixed-effects model analysis. Data was censored for ITT or OP and the rate per 52 weeks is given. F) The mean difference in slope at 52 weeks for Control versus Treatment CD4 trajectories with the 95% confidence intervals are reported. Both ITT and OP censoring are reported with p-values.
