# Supplementary material for: A randomized control trial of high-dose micronutrient-antioxidant supplementation in healthy persons with untreated HIV infection
Source: PLoS One. 2022 Jul 14;17(7):e0270590. doi: 10.1371/journal.pone.0270590 (PMC9282469; doi:10.1371/journal.pone.0270590)
Supplement: S5 Fig — The mean slope for CD4, CD8 and the CD4:CD8 ratio were calculated for Control and Treatment groups (all data was censored for those off-protocol (OP)). A) The difference in the mean slopes for Control versus Treatment are presented for CD4 and CD8 T lymphocytes over 52 weeks with 95% confidence interval (capped bars) and p value. B) The difference in the mean slopes for the CD4:CD8 ratio over 52 weeks is reported with 95% confidence interval (capped bars) and p value. (PPTX) [file pone.0270590.s006.pptx]

## Slide 1
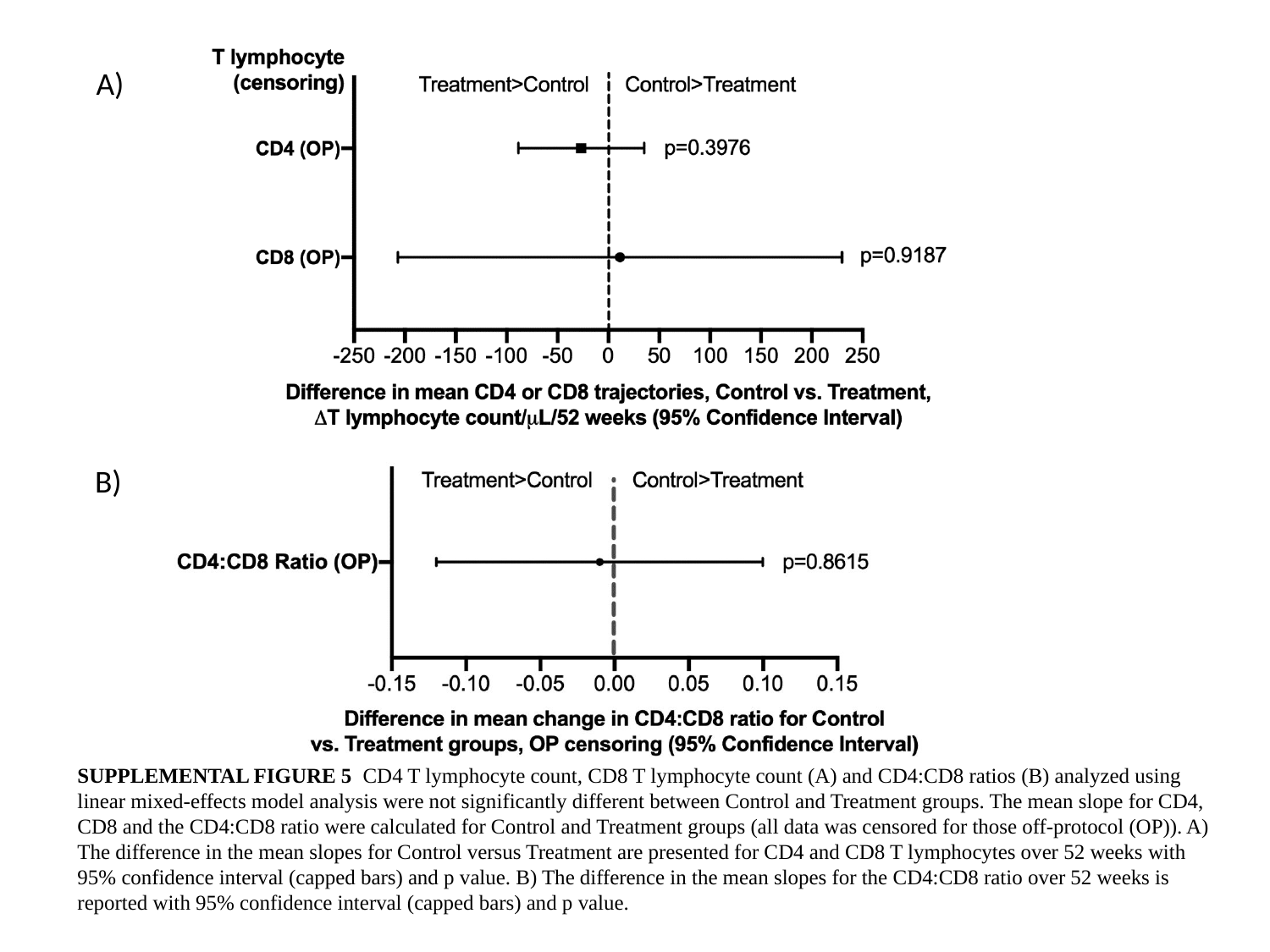

A)
B)
SUPPLEMENTAL FIGURE 5 CD4 T lymphocyte count, CD8 T lymphocyte count (A) and CD4:CD8 ratios (B) analyzed using linear mixed-effects model analysis were not significantly different between Control and Treatment groups. The mean slope for CD4, CD8 and the CD4:CD8 ratio were calculated for Control and Treatment groups (all data was censored for those off-protocol (OP)). A) The difference in the mean slopes for Control versus Treatment are presented for CD4 and CD8 T lymphocytes over 52 weeks with 95% confidence interval (capped bars) and p value. B) The difference in the mean slopes for the CD4:CD8 ratio over 52 weeks is reported with 95% confidence interval (capped bars) and p value.
