# Supplementary material for: A randomized control trial of high-dose micronutrient-antioxidant supplementation in healthy persons with untreated HIV infection
Source: PLoS One. 2022 Jul 14;17(7):e0270590. doi: 10.1371/journal.pone.0270590 (PMC9282469; doi:10.1371/journal.pone.0270590)
Supplement: S6 Fig — HIV viral load of HIV-infected participants on 100% RDA (Control- A, C) versus high-dose (Treatment-B, D) supplements over time, confined to intent-to-treat analysis (ITT, data censored for those starting ART; A, B) or off-protocol censoring (OP; C, D). For graphs A-D, each line represents an individual participant’s measurements taken every 12 weeks until the end of the study (96 weeks or discontinuation of study) and only data from those participants with measurements for at least 36 weeks (i.e. having at least 3 data points) were included. Measurements at Week 0 represent the participant’s baseline HIV viral load prior to taking the indicated supplement. E) Linear mixed-effect model analysis was used to calculate the mean change in HIV viral load (Log10 copies/mL) over time (in Weeks) for Control and Treatment groups. Data was censored for ITT or OP and the rate per 52 weeks is given. F) The difference in mean slope for HIV viral load trajectories of Control versus Treatment groups are reported with the 95% confidence interval as the capped bars and p-values listed beside. (PPTX) [file pone.0270590.s007.pptx]

## Slide 1
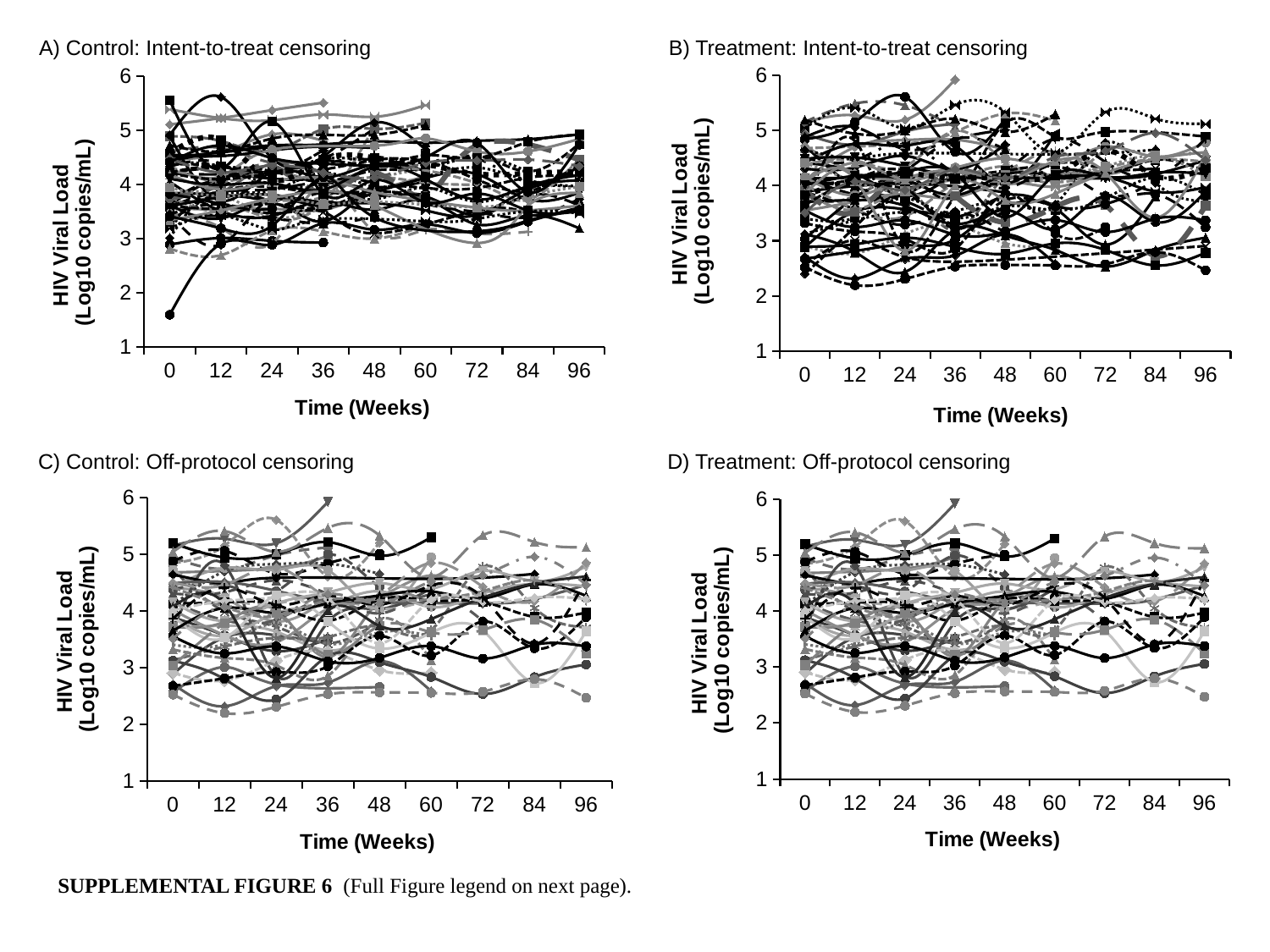

[unsupported chart]
[unsupported chart]
A) Control: Intent-to-treat censoring
B) Treatment: Intent-to-treat censoring
C) Control: Off-protocol censoring
D) Treatment: Off-protocol censoring
[unsupported chart]
[unsupported chart]
SUPPLEMENTAL FIGURE 6 (Full Figure legend on next page).

## Slide 2
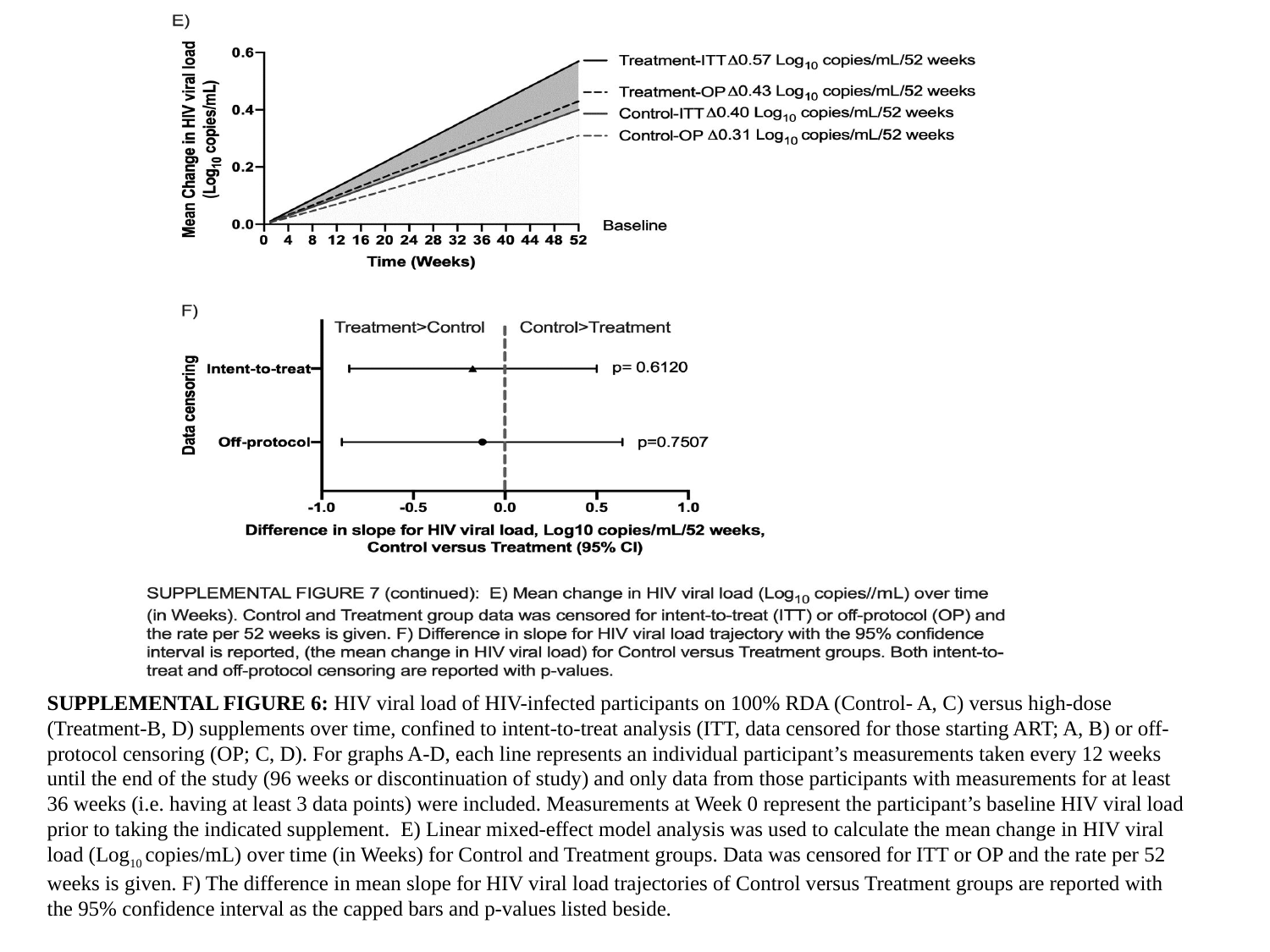

SUPPLEMENTAL FIGURE 6: HIV viral load of HIV-infected participants on 100% RDA (Control- A, C) versus high-dose (Treatment-B, D) supplements over time, confined to intent-to-treat analysis (ITT, data censored for those starting ART; A, B) or off-protocol censoring (OP; C, D). For graphs A-D, each line represents an individual participant’s measurements taken every 12 weeks until the end of the study (96 weeks or discontinuation of study) and only data from those participants with measurements for at least 36 weeks (i.e. having at least 3 data points) were included. Measurements at Week 0 represent the participant’s baseline HIV viral load prior to taking the indicated supplement. E) Linear mixed-effect model analysis was used to calculate the mean change in HIV viral load (Log10 copies/mL) over time (in Weeks) for Control and Treatment groups. Data was censored for ITT or OP and the rate per 52 weeks is given. F) The difference in mean slope for HIV viral load trajectories of Control versus Treatment groups are reported with the 95% confidence interval as the capped bars and p-values listed beside.
