# Supplementary material for: A randomized control trial of high-dose micronutrient-antioxidant supplementation in healthy persons with untreated HIV infection
Source: PLoS One. 2022 Jul 14;17(7):e0270590. doi: 10.1371/journal.pone.0270590 (PMC9282469; doi:10.1371/journal.pone.0270590)
Supplement: S7 Fig — A) Mean slopes for each serum chemistry were calculated for Control and Treatment groups using linear mixed-effects model analysis. B) The difference in mean slope for serum chemistry trajectories of Control versus Treatment groups are reported in change in units (given for each serum chemistry) per 52 weeks, with the 95% confidence interval being reported as the capped bars and p values listed beside. (PPTX) [file pone.0270590.s008.pptx]

## Slide 1
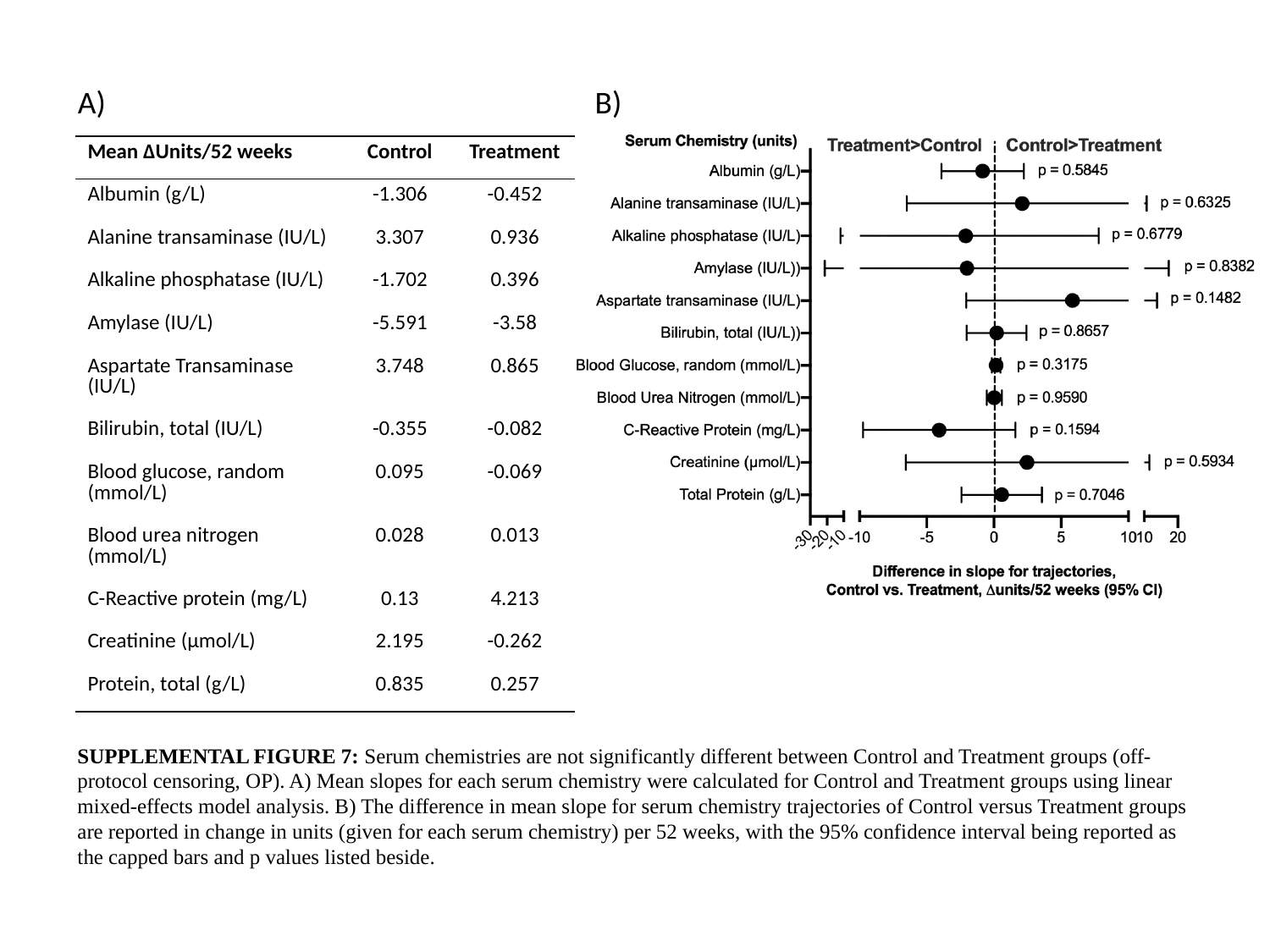

A)
B)
| Mean ΔUnits/52 weeks | Control | Treatment |
| --- | --- | --- |
| Albumin (g/L) | -1.306 | -0.452 |
| Alanine transaminase (IU/L) | 3.307 | 0.936 |
| Alkaline phosphatase (IU/L) | -1.702 | 0.396 |
| Amylase (IU/L) | -5.591 | -3.58 |
| Aspartate Transaminase (IU/L) | 3.748 | 0.865 |
| Bilirubin, total (IU/L) | -0.355 | -0.082 |
| Blood glucose, random (mmol/L) | 0.095 | -0.069 |
| Blood urea nitrogen (mmol/L) | 0.028 | 0.013 |
| C-Reactive protein (mg/L) | 0.13 | 4.213 |
| Creatinine (μmol/L) | 2.195 | -0.262 |
| Protein, total (g/L) | 0.835 | 0.257 |
SUPPLEMENTAL FIGURE 7: Serum chemistries are not significantly different between Control and Treatment groups (off-protocol censoring, OP). A) Mean slopes for each serum chemistry were calculated for Control and Treatment groups using linear mixed-effects model analysis. B) The difference in mean slope for serum chemistry trajectories of Control versus Treatment groups are reported in change in units (given for each serum chemistry) per 52 weeks, with the 95% confidence interval being reported as the capped bars and p values listed beside.
