# Supplementary material for: A randomized control trial of high-dose micronutrient-antioxidant supplementation in healthy persons with untreated HIV infection
Source: PLoS One. 2022 Jul 14;17(7):e0270590. doi: 10.1371/journal.pone.0270590 (PMC9282469; doi:10.1371/journal.pone.0270590)
Supplement: S8 Fig — Participants were asked to grade their QOL at baseline and each study visit (every 12 weeks up to 96 weeks) using the Euro-QoL 5 dimensions—3 level questionnaire. The parameters of activity, anxiety, mobility, pain and self-care were graded as no problem (1), some problems (2) or extreme problems (3). The bars represent the percentage of individuals with no problems in the Control (black bars) versus Treatment (gray bars) groups for (A) Activity, (B) Anxiety, (C) Mobility, (D) Pain, and (E) Self-Care (out of 100% total). Data was censored for participants off-protocol. The n values for Control and Treatment groups at each time point are listed in the table below the respective graph. No statistics were calculated, as these are a change in proportion rather than individual changes over time. (PPTX) [file pone.0270590.s009.pptx]

## Slide 1
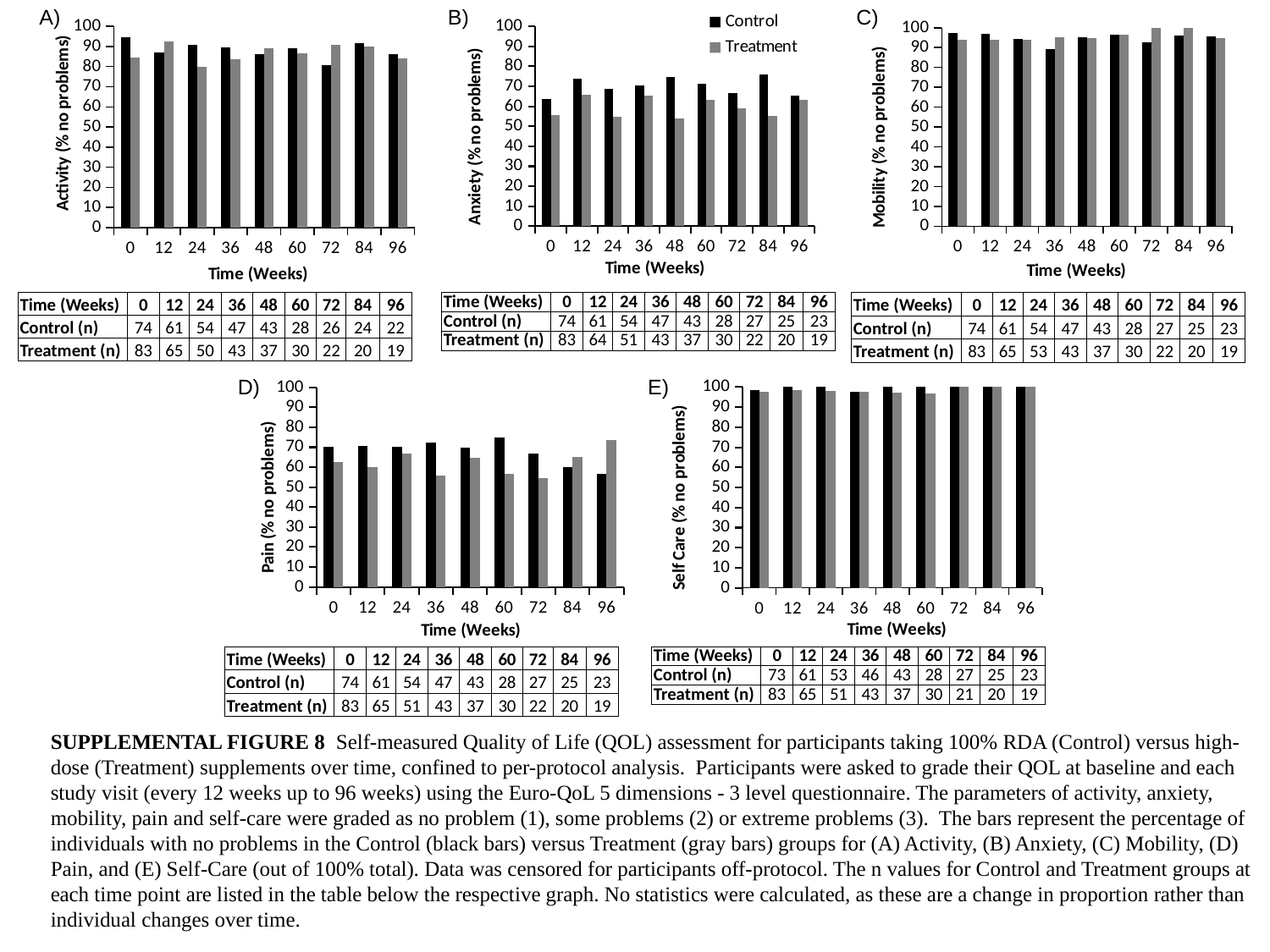

### Chart
| Category | | |
|---|---|---|
| 0 | 63.51351351351352 | 55.42168674698795 |
| 12 | 73.77049180327865 | 65.62499999999999 |
| 24 | 68.51851851851848 | 54.90196078431372 |
| 36 | 70.21276595744678 | 65.1162790697675 |
| 48 | 74.4186046511628 | 54.05405405405399 |
| 60 | 71.42857142857135 | 63.33333333333334 |
| 72 | 66.66666666666667 | 59.09090909090908 |
| 84 | 76.0 | 55.0 |
| 96 | 65.21739130434781 | 63.15789473684199 |
### Chart
| Category | | |
|---|---|---|
| 0 | 94.59459459459458 | 84.33734939759016 |
| 12 | 86.88524590163931 | 92.30769230769228 |
| 24 | 90.74074074074073 | 80.0 |
| 36 | 89.36170212765917 | 83.7209302325578 |
| 48 | 86.046511627907 | 89.18918918918918 |
| 60 | 89.2857142857143 | 86.66666666666667 |
| 72 | 80.7692307692308 | 90.9090909090909 |
| 84 | 91.66666666666667 | 90.0 |
| 96 | 86.36363636363625 | 84.21052631578948 |
### Chart
| Category | | |
|---|---|---|
| 0 | 97.2972972972973 | 93.9759036144578 |
| 12 | 96.7213114754098 | 93.84615384615384 |
| 24 | 94.4444444444445 | 94.11764705882351 |
| 36 | 89.36170212765917 | 95.34883720930233 |
| 48 | 95.34883720930233 | 94.59459459459458 |
| 60 | 96.42857142857135 | 96.66666666666667 |
| 72 | 92.59259259259237 | 100.0 |
| 84 | 96.0 | 100.0 |
| 96 | 95.65217391304328 | 94.73684210526315 |A)
B)
C)
| Time (Weeks) | 0 | 12 | 24 | 36 | 48 | 60 | 72 | 84 | 96 |
| --- | --- | --- | --- | --- | --- | --- | --- | --- | --- |
| Control (n) | 74 | 61 | 54 | 47 | 43 | 28 | 26 | 24 | 22 |
| Treatment (n) | 83 | 65 | 50 | 43 | 37 | 30 | 22 | 20 | 19 |
| Time (Weeks) | 0 | 12 | 24 | 36 | 48 | 60 | 72 | 84 | 96 |
| --- | --- | --- | --- | --- | --- | --- | --- | --- | --- |
| Control (n) | 74 | 61 | 54 | 47 | 43 | 28 | 27 | 25 | 23 |
| Treatment (n) | 83 | 64 | 51 | 43 | 37 | 30 | 22 | 20 | 19 |
| Time (Weeks) | 0 | 12 | 24 | 36 | 48 | 60 | 72 | 84 | 96 |
| --- | --- | --- | --- | --- | --- | --- | --- | --- | --- |
| Control (n) | 74 | 61 | 54 | 47 | 43 | 28 | 27 | 25 | 23 |
| Treatment (n) | 83 | 65 | 53 | 43 | 37 | 30 | 22 | 20 | 19 |
### Chart
| Category | | |
|---|---|---|
| 0 | 98.63013698630135 | 97.59036144578313 |
| 12 | 100.0 | 98.46153846153847 |
| 24 | 100.0 | 98.0392156862745 |
| 36 | 97.87234042553158 | 97.67441860465092 |
| 48 | 100.0 | 97.2972972972973 |
| 60 | 100.0 | 96.66666666666667 |
| 72 | 100.0 | 100.0 |
| 84 | 100.0 | 100.0 |
| 96 | 100.0 | 100.0 |D)
E)
### Chart
| Category | | |
|---|---|---|
| 0 | 70.27027027027027 | 62.65060240963838 |
| 12 | 70.49180327868852 | 60.0 |
| 24 | 70.37037037037014 | 66.66666666666664 |
| 36 | 72.3404255319149 | 55.81395348837209 |
| 48 | 69.76744186046511 | 64.86486486486487 |
| 60 | 75.0 | 56.66666666666644 |
| 72 | 66.66666666666664 | 54.54545454545454 |
| 84 | 60.0 | 65.0 |
| 96 | 56.52173913043478 | 73.6842105263158 || Time (Weeks) | 0 | 12 | 24 | 36 | 48 | 60 | 72 | 84 | 96 |
| --- | --- | --- | --- | --- | --- | --- | --- | --- | --- |
| Control (n) | 74 | 61 | 54 | 47 | 43 | 28 | 27 | 25 | 23 |
| Treatment (n) | 83 | 65 | 51 | 43 | 37 | 30 | 22 | 20 | 19 |
| Time (Weeks) | 0 | 12 | 24 | 36 | 48 | 60 | 72 | 84 | 96 |
| --- | --- | --- | --- | --- | --- | --- | --- | --- | --- |
| Control (n) | 73 | 61 | 53 | 46 | 43 | 28 | 27 | 25 | 23 |
| Treatment (n) | 83 | 65 | 51 | 43 | 37 | 30 | 21 | 20 | 19 |
SUPPLEMENTAL FIGURE 8 Self-measured Quality of Life (QOL) assessment for participants taking 100% RDA (Control) versus high-dose (Treatment) supplements over time, confined to per-protocol analysis. Participants were asked to grade their QOL at baseline and each study visit (every 12 weeks up to 96 weeks) using the Euro-QoL 5 dimensions - 3 level questionnaire. The parameters of activity, anxiety, mobility, pain and self-care were graded as no problem (1), some problems (2) or extreme problems (3). The bars represent the percentage of individuals with no problems in the Control (black bars) versus Treatment (gray bars) groups for (A) Activity, (B) Anxiety, (C) Mobility, (D) Pain, and (E) Self-Care (out of 100% total). Data was censored for participants off-protocol. The n values for Control and Treatment groups at each time point are listed in the table below the respective graph. No statistics were calculated, as these are a change in proportion rather than individual changes over time.
