# Supplementary material for: A randomized control trial of high-dose micronutrient-antioxidant supplementation in healthy persons with untreated HIV infection
Source: PLoS One. 2022 Jul 14;17(7):e0270590. doi: 10.1371/journal.pone.0270590 (PMC9282469; doi:10.1371/journal.pone.0270590)
Supplement: S9 Fig — (PPTX) [file pone.0270590.s010.pptx]

## Slide 1
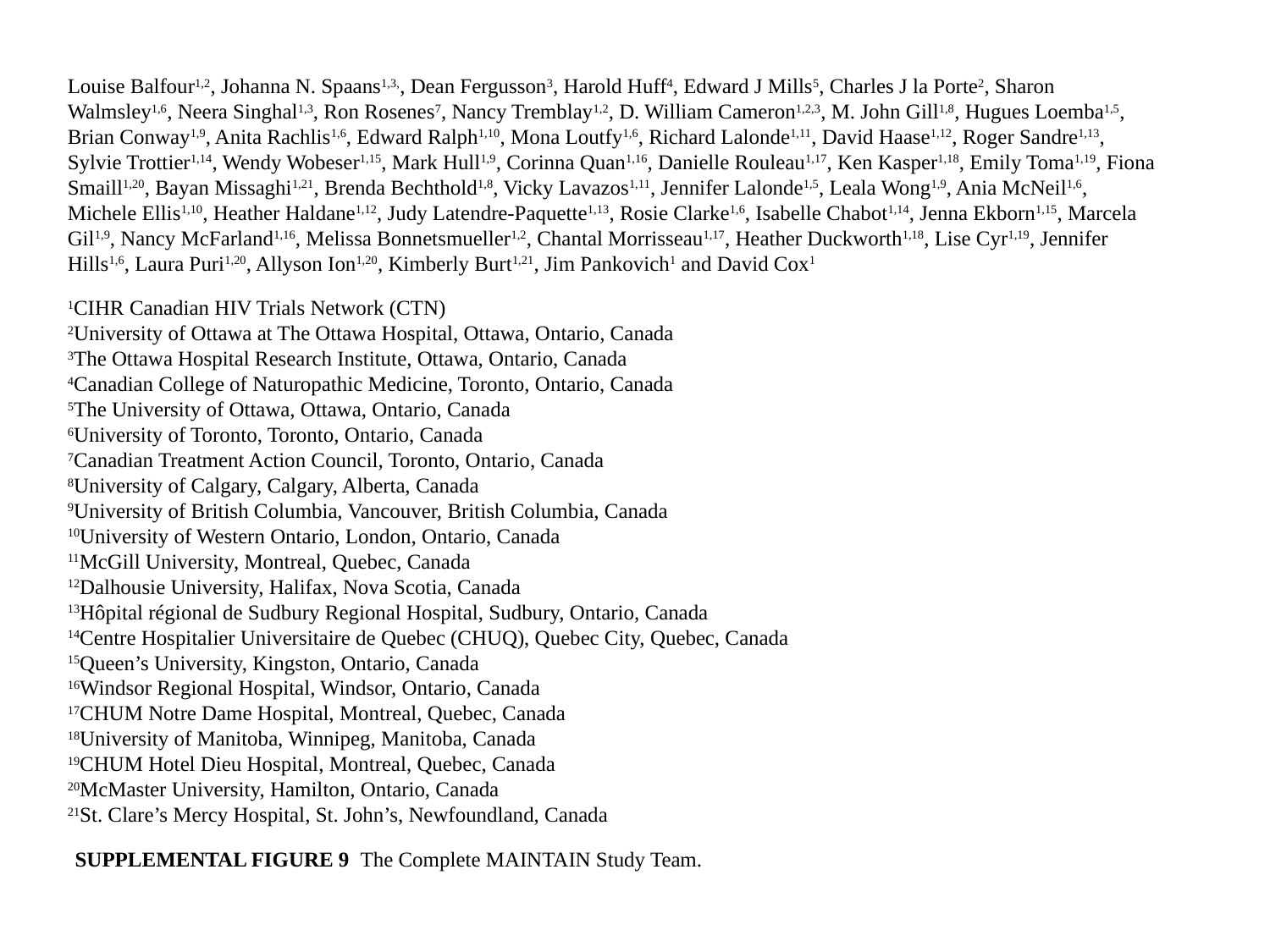

Louise Balfour1,2, Johanna N. Spaans1,3,, Dean Fergusson3, Harold Huff4, Edward J Mills5, Charles J la Porte2, Sharon Walmsley1,6, Neera Singhal1,3, Ron Rosenes7, Nancy Tremblay1,2, D. William Cameron1,2,3, M. John Gill1,8, Hugues Loemba1,5, Brian Conway1,9, Anita Rachlis1,6, Edward Ralph1,10, Mona Loutfy1,6, Richard Lalonde1,11, David Haase1,12, Roger Sandre1,13, Sylvie Trottier1,14, Wendy Wobeser1,15, Mark Hull1,9, Corinna Quan1,16, Danielle Rouleau1,17, Ken Kasper1,18, Emily Toma1,19, Fiona Smaill1,20, Bayan Missaghi1,21, Brenda Bechthold1,8, Vicky Lavazos1,11, Jennifer Lalonde1,5, Leala Wong1,9, Ania McNeil1,6, Michele Ellis1,10, Heather Haldane1,12, Judy Latendre-Paquette1,13, Rosie Clarke1,6, Isabelle Chabot1,14, Jenna Ekborn1,15, Marcela Gil1,9, Nancy McFarland1,16, Melissa Bonnetsmueller1,2, Chantal Morrisseau1,17, Heather Duckworth1,18, Lise Cyr1,19, Jennifer Hills1,6, Laura Puri1,20, Allyson Ion1,20, Kimberly Burt1,21, Jim Pankovich1 and David Cox1
1CIHR Canadian HIV Trials Network (CTN)
2University of Ottawa at The Ottawa Hospital, Ottawa, Ontario, Canada
3The Ottawa Hospital Research Institute, Ottawa, Ontario, Canada
4Canadian College of Naturopathic Medicine, Toronto, Ontario, Canada
5The University of Ottawa, Ottawa, Ontario, Canada
6University of Toronto, Toronto, Ontario, Canada
7Canadian Treatment Action Council, Toronto, Ontario, Canada
8University of Calgary, Calgary, Alberta, Canada
9University of British Columbia, Vancouver, British Columbia, Canada
10University of Western Ontario, London, Ontario, Canada
11McGill University, Montreal, Quebec, Canada
12Dalhousie University, Halifax, Nova Scotia, Canada
13Hôpital régional de Sudbury Regional Hospital, Sudbury, Ontario, Canada
14Centre Hospitalier Universitaire de Quebec (CHUQ), Quebec City, Quebec, Canada
15Queen’s University, Kingston, Ontario, Canada
16Windsor Regional Hospital, Windsor, Ontario, Canada
17CHUM Notre Dame Hospital, Montreal, Quebec, Canada
18University of Manitoba, Winnipeg, Manitoba, Canada
19CHUM Hotel Dieu Hospital, Montreal, Quebec, Canada
20McMaster University, Hamilton, Ontario, Canada
21St. Clare’s Mercy Hospital, St. John’s, Newfoundland, Canada
SUPPLEMENTAL FIGURE 9 The Complete MAINTAIN Study Team.
