# Supplementary material for: A randomized control trial of high-dose micronutrient-antioxidant supplementation in healthy persons with untreated HIV infection
Source: PLoS One. 2022 Jul 14;17(7):e0270590. doi: 10.1371/journal.pone.0270590 (PMC9282469; doi:10.1371/journal.pone.0270590)
Supplement: S1 Table — Treatment refers to the High-dose supplement and Control refers to the 100% recommended daily allowance supplement. The readings are given in different units based on the micronutrient per 16 capsules (daily-dose given to participants). (DOCX) [file pone.0270590.s011.docx]

**SUPPLEMENTAL TABLE 1** Supplement Stability Report per 16 capsules (actual daily dose) over the course of the study. Treatment refers to the High-dose supplement and Control refers to the 100% recommended daily allowance supplement. The readings are given in different units based on the micronutrient per 16 capsules (daily-dose given to participants).

| Date (mm/dd/year) | Vitamin B_6_ (mg) | Carotenoids (IU) | Vitamin C (mg) | Iron (mg) | Selenium (µg) | Zinc (mg) | Acetyl-L-Carnitine (mg) | Lipoic Acid (mg) | n-Acetyl Cysteine (mg) |
| --- | --- | --- | --- | --- | --- | --- | --- | --- | --- |
| **Control^1.2^** | **2** | **3,500** | **60** | **18** | **20** | **15** | **0** | **0** | **0** |
| 12/22/2008 | - | 3,388 | 57.88 | 19.96 | 19.32 | 19.40 |  |  |  |
| 07/22/2009 | - | - | - | - | - | - |  |  |  |
| 01/06/2010 | 4.04 | 5,636 | 81.84 | 15.52 | * | 17.52 |  |  |  |
| 07/27/2010 | 3.84 | 4,000 | 62.20 | - | - | - |  |  |  |
| 10/12/2010 | - | 4,960 | 9.60 | - | 20.80 | - |  |  |  |
| 08/10/2011 | - | - | 65.52 | 18.24 | - | 14.40 |  |  |  |
| 06/13/2012 | 3.72 | - | 61.28 | - | 25.24 | 14.64 |  |  |  |
| 11/26/2012 | - | - | 48.84 | 23.12 | - | 13.88 |  |  |  |
| **Treatment^1,3^** | **200** | **20,000** | **2,000** | **18** | **200** | **30** | **1,000** | **400** | **1,200** |
| 12/22/2008 | 229.16 | 31,028 | 1,008 | - | 267.68 | 24.32 | 1,304 | 382.16 | 1,296 |
| 7/22/2009 | 262.64 | 26,876 | 2,352 | - | - | 35.40 | 980 | 314.60 | - |
| 06/06/2010 | 261.36 | 18,788 | 2,320 | - | - | 38.96 | 1,080 | - | 1,428 |
| 07/27/2010 | 276.28 | 14,444 | 2,224 | - | - | - | 1,132 | 400.00 | 1,264 |
| 10/12/2010 | 257.52 | 40,788 | 1,696 | - | 378.76 | - | 1,016 | 364.32 | 1,296 |
| 08/10/2011 | 265.24 | 18,932 | - | - | 297.24 | 36.08 | 1,120 | 416.00 | 1,392 |
| 06/13/2012 | 260.56 | 14,760 | 2,388 | - | 217.08 | 30.92 | 1,008 | 420.00 | 1,332 |
| 11/26/2012 | - | 20,128 | 2,236 | - | 258.68 | 35.20 | 1,024 | 480.00 | 1,220 |

^1^The readings are given in different units based on the micronutrient per 16 capsules (daily dose was 8 capsules taken twice a day for 96 weeks). Original amount of each micronutrient as reported by the manufacturer’s label for the daily dose of 16 capsules (14).

^2^Control refers to the 100% recommended daily allowance supplement.

^3^Treatment refers to the High-dose supplement.

*Reading taken, but was out of range.
