# Supplementary material for: A randomized control trial of high-dose micronutrient-antioxidant supplementation in healthy persons with untreated HIV infection
Source: PLoS One. 2022 Jul 14;17(7):e0270590. doi: 10.1371/journal.pone.0270590 (PMC9282469; doi:10.1371/journal.pone.0270590)
Supplement: S2 Table — Note that the 29 serious adverse events happened to 19 individual participants. All the serious adverse events were reported to Health Canada and to the local REB. All were deemed unexpected and unrelated to the study treatment. (DOCX) [file pone.0270590.s012.docx]

**SUPPLEMENTAL TABLE 2** Serious Adverse Events among MAINTAIN participants. Note that the 29 serious adverse events happened to 19 individual participants. All the serious adverse events were reported to Health Canada and to the local REB. All were deemed unexpected and unrelated to the study treatment.

| **Serious Adverse Events** | **Total (n)** |
| --- | --- |
| Appendicitis | 2 |
| Community-acquired pneumonia | 2 |
| Fever and Lymphadenopathy | 2 |
| Intra abdominal abscess | 2 |
| Metastatic renal cell carcinoma | 2 |
| Lymphoma^1^ | 2 |
| Attempted suicide | 1 |
| Diverticulitis Hospitalization | 1 |
| Drug overdose | 1 |
| Drug rash | 1 |
| Hepatitis C | 1 |
| Hospitalization due to psychosis | 1 |
| Hospitalization due to splenomegaly | 1 |
| Hospitalization due to depression | 1 |
| Hospitalization due to suicide attempt | 1 |
| Infection-diarrhea | 1 |
| Laparoscopic Cholecystectomy | 1 |
| Lorzepam overdose | 1 |
| Perianal swelling | 1 |
| Perianal abscess | 1 |
| Right elbow septic arthritis | 1 |
| Right leg cellulitis | 1 |
| Suppurative cholecystitis | 1 |
| **Total Serious Adverse Events** | **29** |

^1^Of the two Lymphoma cases reported, one was biopsy proven and the other case was suspected but was lost to follow up. Both were from the treatment group, however both were off-protocol due to already reaching an outcome prior to being diagnosed. Therefore, neither case is considered to be a primary outcome.

In the first lymphoma case, the participant had started ART after 84 weeks and was diagnosed with Stage IIA Non-Hodgkins lymphoma at the 96-week visit, about 661 days after the first dose.

In the other lymphoma case, the participant withdrew prior to the 12-week visit due to pill burden. The off-protocol participant was diagnosed with a suspected lymphoma 91 days after their first dose and was subsequently treated with ART (Atripla).
