# Supplementary material for: A randomized control trial of high-dose micronutrient-antioxidant supplementation in healthy persons with untreated HIV infection
Source: PLoS One. 2022 Jul 14;17(7):e0270590. doi: 10.1371/journal.pone.0270590 (PMC9282469; doi:10.1371/journal.pone.0270590)
Supplement: S3 Table — (DOCX) [file pone.0270590.s013.docx]

**SUPPLEMENTAL TABLE 3**  Albumin measurements (in blood) taken quarterly over the study period in Control (100% recommended daily allowance supplement) and Treatment (High-dose supplement) groups.

|  | Time (Weeks) | Median  (g/L) | Mean ± SD  (g/L) | n | % Frequency Low^2,3^ |
| --- | --- | --- | --- | --- | --- |
| Control^1^ | 0 | 43.0 | 45.67 ± 35.97 | 75 | 1.33 |
|  | 12 | 42.0 | 41.95 ± 4.17 | 58 | 0.00 |
|  | 24 | 43.0 | 42.50 ± 3.28 | 54 | 0.00 |
|  | 36 | 43.0 | 42.38 ± 3.46 | 47 | 0.00 |
|  | 48 | 44.0 | 43.15 ± 3.62 | 39 | 0.00 |
|  | 60 | 43.0 | 42.07 ± 3.41 | 27 | 0.00 |
|  | 72 | 42.0 | 42.31 ± 3.56 | 26 | 0.00 |
|  | 84 | 42.0 | 42.75 ± 3.18 | 24 | 0.00 |
|  | 96 | 43.0 | 43.05 ± 3.57 | 22 | 0.00 |
| Treatment^1^ | 0 | 42.0 | 45.34 ± 30.78 | 83 | 2.41 |
|  | 12 | 42.5 | 42.20 ± 4.24 | 64 | 1.56 |
|  | 24 | 43.0 | 42.86 ± 3.64 | 51 | 0.00 |
|  | 36 | 43.0 | 42.37 ± 4.32 | 41 | 2.44 |
|  | 48 | 43.0 | 42.19 ± 4.19 | 32 | 3.13 |
|  | 60 | 43.0 | 42.27 ± 3.94 | 30 | 6.67 |
|  | 72 | 42.0 | 41.62 ± 3.75 | 21 | 0.00 |
|  | 84 | 42.0 | 41.74 ± 3.75 | 19 | 0.00 |
|  | 96 | 42.0 | 41.50 ± 3.61 | 18 | 0.00 |

^1^Data was censored for those participants off-protocol.

^2^Normal Range for Albumin in blood is 34-50 g/L (as per Eastern Ontario Regional Laboratory Association normal reference range).

^3^Percentage (%) Frequency Low refers to number of times a reading was less than 34 g/L normalized to the number (n) of total readings at that time point.
