# Supplementary material for: A randomized control trial of high-dose micronutrient-antioxidant supplementation in healthy persons with untreated HIV infection
Source: PLoS One. 2022 Jul 14;17(7):e0270590. doi: 10.1371/journal.pone.0270590 (PMC9282469; doi:10.1371/journal.pone.0270590)
Supplement: S4 Table — (DOCX) [file pone.0270590.s014.docx]

**SUPPLEMENTAL TABLE 4** Alanine Transaminase (ALT) measurements (in blood) taken quarterly over the study period in Control (100% recommended daily allowance supplement) and Treatment (High-dose supplement) groups.

|  | Time (Weeks) | Median  (IU/L) | Mean ± SD  (IU/L) | n | % Frequency High^2,3^ |
| --- | --- | --- | --- | --- | --- |
| Control^1^ | 0 | 22.0 | 25.80 ± 12.96 | 76 | 1.32 |
|  | 12 | 25.0 | 29.07 ± 15.57 | 61 | 3.28 |
|  | 24 | 26.0 | 28.30 ± 14.21 | 54 | 3.70 |
|  | 36 | 24.0 | 30.52 ± 16.40 | 48 | 6.25 |
|  | 48 | 26.0 | 33.15 ± 20.86 | 41 | 7.32 |
|  | 60 | 27.0 | 30.33 ± 14.94 | 27 | 0.00 |
|  | 72 | 25.5 | 30.42 ± 17.80 | 26 | 7.69 |
|  | 84 | 22.0 | 27.58 ± 14.75 | 24 | 8.33 |
|  | 96 | 25.5 | 30.95 ± 16.51 | 22 | 4.55 |
| Treatment^1^ | 0 | 25.0 | 30.71 ± 20.53 | 83 | 6.02 |
|  | 12 | 30.0 | 39.68 ± 37.51 | 65 | 7.69 |
|  | 24 | 28.0 | 34.91 ± 22.11 | 53 | 7.55 |
|  | 36 | 28.0 | 34.44 ± 19.38 | 43 | 6.98 |
|  | 48 | 30.0 | 35.22 ± 18.92 | 37 | 5.41 |
|  | 60 | 31.0 | 33.16 ± 17.20 | 31 | 6.45 |
|  | 72 | 25.0 | 32.00 ± 17.30 | 22 | 9.09 |
|  | 84 | 31.0 | 37.00 ± 23.50 | 20 | 5.00 |
|  | 96 | 29.0 | 37.37 ± 26.32 | 19 | 5.26 |

^1^Data was censored for those participants off-protocol.

^2^Normal Range for ALT in blood is 17-63 IU/L (as per Eastern Ontario Regional Laboratory Association normal reference range).

^3^Percentage (%) Frequency High refers to number of times a reading was greater than 63 IU/L normalized to the number (n) of total readings at that time point.
