# Supplementary material for: A randomized control trial of high-dose micronutrient-antioxidant supplementation in healthy persons with untreated HIV infection
Source: PLoS One. 2022 Jul 14;17(7):e0270590. doi: 10.1371/journal.pone.0270590 (PMC9282469; doi:10.1371/journal.pone.0270590)
Supplement: S5 Table — (DOCX) [file pone.0270590.s015.docx]

**SUPPLEMENTAL TABLE 5** Alkaline Phosphatase measurements (in blood) taken quarterly over the study period in Control (100% recommended daily allowance supplement) and Treatment (High-dose supplement) groups.

|  | Time (Weeks) | Median  (IU/L) | Mean ± SD  (IU/L) | n | % Frequency High^2,3^ |
| --- | --- | --- | --- | --- | --- |
| Control^1^ | 0 | 67.5 | 70.09 ± 20.36 | 76 | 2.63 |
|  | 12 | 67.5 | 71.74 ± 20.28 | 62 | 3.23 |
|  | 24 | 66.0 | 70.55 ± 22.21 | 55 | 1.82 |
|  | 36 | 65.0 | 68.09 ± 18.32 | 47 | 2.13 |
|  | 48 | 68.0 | 70.22 ± 27.55 | 41 | 2.44 |
|  | 60 | 71.0 | 77.37 ± 32.23 | 27 | 3.70 |
|  | 72 | 68.0 | 71.52 ± 30.81 | 27 | 3.70 |
|  | 84 | 64.0 | 71.72 ± 30.46 | 25 | 4.00 |
|  | 96 | 64.0 | 70.74 ± 31.49 | 23 | 4.35 |
| Treatment^1^ | 0 | 70.0 | 70.29 ± 18.71 | 83 | 0.00 |
|  | 12 | 70.0 | 68.74 ± 17.03 | 65 | 0.00 |
|  | 24 | 69.0 | 68.49 ± 16.60 | 55 | 0.00 |
|  | 36 | 68.0 | 69.51 ± 17.21 | 43 | 0.00 |
|  | 48 | 70.5 | 74.06 ± 21.06 | 36 | 0.00 |
|  | 60 | 71.0 | 73.84 ± 18.05 | 31 | 0.00 |
|  | 72 | 67.5 | 72.86 ± 21.59 | 22 | 0.00 |
|  | 84 | 65.5 | 70.85 ± 19.79 | 20 | 0.00 |
|  | 96 | 70.0 | 72.00 ± 19.78 | 19 | 0.00 |

^1^Data was censored for those participants off-protocol.

^2^Normal Range for alkaline phosphatase in blood is 50-136 IU/L (as per Eastern Ontario Regional Laboratory Association normal reference range).

^3^Percentage (%) Frequency High refers to number of times a reading was more than 136 IU/L normalized to the number (n) of total readings at that time point.
