# Supplementary material for: A randomized control trial of high-dose micronutrient-antioxidant supplementation in healthy persons with untreated HIV infection
Source: PLoS One. 2022 Jul 14;17(7):e0270590. doi: 10.1371/journal.pone.0270590 (PMC9282469; doi:10.1371/journal.pone.0270590)
Supplement: S6 Table — (DOCX) [file pone.0270590.s016.docx]

**SUPPLEMENTAL TABLE 6**  Amylase measurements (in blood) taken quarterly over the study period in Control (100% recommended daily allowance supplement) and Treatment (High-dose supplement) groups.

|  | Time (Weeks) | Median  (IU/L) | Mean ± SD  (IU/L) | n | % Frequency High^2,3^ |
| --- | --- | --- | --- | --- | --- |
| Control^1^ | 0 | 71.0 | 72.07 ± 21.32 | 41 | 2.44 |
|  | 12 | 73.0 | 72.24 ± 21.91 | 25 | 4.00 |
|  | 24 | 65.0 | 73.05 ± 26.90 | 21 | 14.29 |
|  | 36 | 59.0 | 67.11 ± 25.16 | 19 | 5.26 |
|  | 48 | 66.5 | 71.21 ± 22.41 | 14 | 7.14 |
|  | 60 | 59.0 | 88.33 ± 82.45 | 9 | 11.11 |
|  | 72 | 60.5 | 66.30 ± 18.43 | 10 | 0.00 |
|  | 84 | 60.5 | 62.00 ± 16.76 | 10 | 0.00 |
|  | 96 | 66.0 | 80.56 ± 46.35 | 9 | 11.11 |
| Treatment^1^ | 0 | 57.0 | 65.55 ± 30.13 | 44 | 9.09 |
|  | 12 | 65.0 | 68.07 ± 27.94 | 27 | 11.11 |
|  | 24 | 59.0 | 64.83 ± 27.73 | 23 | 4.35 |
|  | 36 | 57.0 | 71.28 ± 44.28 | 18 | 16.67 |
|  | 48 | 60.0 | 64.15 ± 20.34 | 13 | 0.00 |
|  | 60 | 60.0 | 62.56 ± 11.91 | 9 | 0.00 |
|  | 72 | 67.5 | 67.75 ± 13.24 | 8 | 0.00 |
|  | 84 | 63.0 | 56.43 ± 11.31 | 7 | 0.00 |
|  | 96 | 63.0 | 59.71 ± 16.73 | 7 | 0.00 |

^1^Data was censored for those participants off-protocol.

^2^Normal Range for amylase in blood is 25-115 IU/L (as per Eastern Ontario Regional Laboratory Association normal reference range).

^3^Percentage (%) Frequency High refers to number of times a reading was more than 115 IU/L normalized to the number (n) of total readings at that time point.
