# Supplementary material for: A randomized control trial of high-dose micronutrient-antioxidant supplementation in healthy persons with untreated HIV infection
Source: PLoS One. 2022 Jul 14;17(7):e0270590. doi: 10.1371/journal.pone.0270590 (PMC9282469; doi:10.1371/journal.pone.0270590)
Supplement: S7 Table — (DOCX) [file pone.0270590.s017.docx]

**SUPPLEMENTAL TABLE 7**  Aspartate Transaminase (AST) measurements (in blood) taken quarterly over the study period in Control (100% recommended daily allowance supplement) and Treatment (High-dose supplement) groups.

|  | Time (Weeks) | Median  (IU/L) | Mean ± SD  (IU/L) | n | % Frequency High^2,3^ |
| --- | --- | --- | --- | --- | --- |
| Control^1^ | 0 | 22.0 | 23.50 ± 6.82 | 76 | 3.95 |
|  | 12 | 24.0 | 25.92 ± 9.66 | 62 | 8.06 |
|  | 24 | 24.0 | 26.39 ± 9.00 | 54 | 7.41 |
|  | 36 | 24.0 | 26.68 ± 8.79 | 47 | 10.64 |
|  | 48 | 26.0 | 28.05 ± 10.55 | 41 | 9.76 |
|  | 60 | 27.0 | 29.48 ± 11.82 | 27 | 18.52 |
|  | 72 | 25.5 | 28.31 ± 11.22 | 26 | 11.54 |
|  | 84 | 26.0 | 26.44 ± 9.33 | 25 | 4.00 |
|  | 96 | 25.0 | 33.70 ± 23.10 | 23 | 17.39 |
| Treatment^1^ | 0 | 24.5 | 30.30 ± 20.56 | 82 | 14.63 |
|  | 12 | 29.0 | 42.38 ± 89.21 | 65 | 20.00 |
|  | 24 | 31.0 | 32.25 ± 11.32 | 51 | 27.45 |
|  | 36 | 29.0 | 30.10 ± 9.09 | 40 | 25.00 |
|  | 48 | 28.0 | 31.03 ± 16.30 | 36 | 16.67 |
|  | 60 | 28.5 | 33.70 ± 21.56 | 30 | 26.67 |
|  | 72 | 29.0 | 31.71 ± 13.68 | 21 | 19.05 |
|  | 84 | 26.5 | 30.15 ± 14.17 | 20 | 15.00 |
|  | 96 | 31.0 | 33.42 ± 15.22 | 19 | 26.32 |

^1^Data was censored for those participants off-protocol.

^2^Normal Range for AST in blood is 15-37 IU/L (as per Eastern Ontario Regional Laboratory Association normal reference range).

^3^Percentage (%) Frequency High refers to number of times a reading was more than 37 IU/L normalized to the number (n) of total readings at that time point.
