# Supplementary material for: A randomized control trial of high-dose micronutrient-antioxidant supplementation in healthy persons with untreated HIV infection
Source: PLoS One. 2022 Jul 14;17(7):e0270590. doi: 10.1371/journal.pone.0270590 (PMC9282469; doi:10.1371/journal.pone.0270590)
Supplement: S8 Table — (DOCX) [file pone.0270590.s018.docx]

**SUPPLEMENTAL TABLE 8**  Bilirubin (total) measurements (in blood) taken quarterly over the study period in Control (100% recommended daily allowance supplement) and Treatment (High-dose supplement) groups.

|  | Time (Weeks) | Median  (IU/L) | Mean ± SD  (IU/L) | n | % Frequency High^2,3^ |
| --- | --- | --- | --- | --- | --- |
| Control^1^ | 0 | 9.0 | 10.71 ± 8.56 | 76 | 6.58 |
|  | 12 | 8.0 | 9.19 ± 4.33 | 62 | 6.45 |
|  | 24 | 9.0 | 10.18 ± 5.11 | 55 | 7.27 |
|  | 36 | 9.0 | 9.51 ± 4.06 | 47 | 6.38 |
|  | 48 | 9.0 | 10.15 ± 4.98 | 41 | 9.76 |
|  | 60 | 9.0 | 9.04 ± 3.11 | 26 | 0.00 |
|  | 72 | 8.0 | 9.27 ± 4.39 | 26 | 7.69 |
|  | 84 | 11.0 | 10.79 ± 4.29 | 24 | 8.33 |
|  | 96 | 9.0 | 9.52 ± 3.27 | 23 | 0.00 |
| Treatment^1^ | 0 | 9.0 | 10.82 ± 7.01 | 82 | 10.98 |
|  | 12 | 10.0 | 10.65 ± 5.23 | 65 | 13.85 |
|  | 24 | 10.0 | 11.67 ± 9.48 | 52 | 9.62 |
|  | 36 | 11.0 | 11.02 ± 5.04 | 42 | 11.90 |
|  | 48 | 9.0 | 9.73 ± 4.57 | 37 | 8.11 |
|  | 60 | 9.0 | 10.23 ± 4.59 | 31 | 6.45 |
|  | 72 | 7.5 | 9.50 ± 5.45 | 22 | 9.09 |
|  | 84 | 7.5 | 9.35 ± 4.74 | 20 | 15.00 |
|  | 96 | 8.0 | 9.32 ± 4.23 | 19 | 5.26 |

^1^Data was censored for those participants off-protocol.

^2^Normal Range for total Bilirubin in blood is 3.0 – 17.0 IU/L (as per Eastern Ontario Regional Laboratory Association normal reference range).

^3^Percentage (%) Frequency High refers to number of times a reading was more than 17.0 IU/L normalized to the number (n) of total readings at that time point.
