# Supplementary material for: A randomized control trial of high-dose micronutrient-antioxidant supplementation in healthy persons with untreated HIV infection
Source: PLoS One. 2022 Jul 14;17(7):e0270590. doi: 10.1371/journal.pone.0270590 (PMC9282469; doi:10.1371/journal.pone.0270590)
Supplement: S9 Table — (DOCX) [file pone.0270590.s019.docx]

**SUPPLEMENTAL TABLE 9**  Blood Glucose (random) measurements taken quarterly over the study period in Control (100% recommended daily allowance supplement) and Treatment (High-dose supplement) groups.

|  | Time (Weeks) | Median  (mmol/L) | Mean ± SD  (mmol/L) | n |
| --- | --- | --- | --- | --- |
| Control^1,2^ | 0 | 4.90 | 4.96 ± 0.89 | 75 |
|  | 12 | 4.90 | 5.03 ± 0.87 | 60 |
|  | 24 | 4.95 | 4.95 ± 0.78 | 54 |
|  | 36 | 5.10 | 5.21 ± 1.07 | 46 |
|  | 48 | 4.90 | 5.24 ± 1.31 | 41 |
|  | 60 | 4.80 | 5.13 ± 0.87 | 27 |
|  | 72 | 5.00 | 4.96 ± 0.98 | 27 |
|  | 84 | 4.95 | 5.06 ± 0.52 | 24 |
|  | 96 | 5.00 | 5.08 ± 0.59 | 23 |
| Treatment^1,2^ | 0 | 5.00 | 5.04 ± 0.82 | 83 |
|  | 12 | 4.90 | 4.90 ± 0.75 | 65 |
|  | 24 | 4.90 | 4.90 ± 0.85 | 52 |
|  | 36 | 4.75 | 4.77 ± 0.72 | 42 |
|  | 48 | 4.90 | 4.92 ± 0.74 | 37 |
|  | 60 | 5.00 | 4.91 ± 1.01 | 30 |
|  | 72 | 5.05 | 5.05 ± 0.71 | 22 |
|  | 84 | 5.00 | 4.92 ± 1.00 | 20 |
|  | 96 | 4.90 | 4.92 ± 0.57 | 18 |

^1^Data was censored for those participants off-protocol.

^2^Normal Range for random blood glucose is 4.0 – 11.0 mmol/L (as per Eastern Ontario Regional Laboratory Association normal reference range). No readings were above the normal range.
