# Supplementary material for: A randomized control trial of high-dose micronutrient-antioxidant supplementation in healthy persons with untreated HIV infection
Source: PLoS One. 2022 Jul 14;17(7):e0270590. doi: 10.1371/journal.pone.0270590 (PMC9282469; doi:10.1371/journal.pone.0270590)
Supplement: S10 Table — (DOCX) [file pone.0270590.s020.docx]

**SUPPLEMENTAL TABLE 10**  Blood urea nitrogen (BUN) measurements taken quarterly over the study period in Control (100% recommended daily allowance supplement) and Treatment (High-dose supplement) groups.

|  | Time (Weeks) | Median  (mmol/L) | Mean ± SD  (mmol/L) | n | % Frequency High^2,3^ |
| --- | --- | --- | --- | --- | --- |
| Control^1^ | 0 | 4.60 | 4.75 ± 1.40 | 73 | 0.00 |
|  | 12 | 5.00 | 4.92 ± 1.37 | 54 | 0.00 |
|  | 24 | 4.80 | 4.99 ± 1.46 | 52 | 3.85 |
|  | 36 | 4.70 | 4.75 ± 1.41 | 46 | 4.35 |
|  | 48 | 4.85 | 4.78 ± 1.37 | 40 | 0.00 |
|  | 60 | 4.65 | 4.87 ± 1.32 | 26 | 0.00 |
|  | 72 | 4.90 | 5.11 ± 1.13 | 24 | 0.00 |
|  | 84 | 5.10 | 5.10 ± 1.36 | 23 | 4.35 |
|  | 96 | 4.80 | 4.76 ± 1.11 | 21 | 0.00 |
| Treatment^1^ | 0 | 4.75 | 4.94 ± 1.39 | 82 | 0.00 |
|  | 12 | 4.95 | 5.00 ± 1.40 | 64 | 3.13 |
|  | 24 | 4.50 | 4.96 ± 1.50 | 49 | 2.04 |
|  | 36 | 4.80 | 5.04 ± 1.38 | 38 | 5.26 |
|  | 48 | 5.00 | 5.02 ± 1.24 | 32 | 3.13 |
|  | 60 | 5.30 | 5.44 ± 1.18 | 27 | 3.70 |
|  | 72 | 4.80 | 5.10 ± 1.47 | 19 | 0.00 |
|  | 84 | 4.80 | 5.01 ± 1.11 | 19 | 0.00 |
|  | 96 | 5.00 | 5.49 ± 1.57 | 17 | 11.76 |

^1^Data was censored for those participants off-protocol.

^2^Normal Range for BUN is 2.1-8.0 mmol/L (as per Eastern Ontario Regional Laboratory Association normal reference range).

^3^Percentage (%) Frequency High refers to number of times a reading was more than 8.0 mmol/L normalized to the number (n) of total readings at that time point.
