# Supplementary material for: A randomized control trial of high-dose micronutrient-antioxidant supplementation in healthy persons with untreated HIV infection
Source: PLoS One. 2022 Jul 14;17(7):e0270590. doi: 10.1371/journal.pone.0270590 (PMC9282469; doi:10.1371/journal.pone.0270590)
Supplement: S11 Table — (DOCX) [file pone.0270590.s021.docx]

**SUPPLEMENTAL TABLE 11**  C-reactive protein (CRP) measurements (in blood) taken quarterly over the study period in Control (100% recommended daily allowance supplement) and Treatment (High-dose supplement) groups.

|  | Time (Weeks) | Median  (mg/L) | Mean ± SD  (mg/L) | n | % Frequency High^2,3^ |
| --- | --- | --- | --- | --- | --- |
| Control^1^ | 0 | 1.60 | 3.43 ± 5.57 | 71 | 5.63 |
|  | 12 | 1.20 | 3.87 ± 8.01 | 54 | 7.41 |
|  | 24 | 1.20 | 2.81 ± 5.74 | 50 | 4.00 |
|  | 36 | 1.30 | 4.24 ± 10.23 | 42 | 7.14 |
|  | 48 | 1.35 | 4.78 ± 14.20 | 40 | 5.00 |
|  | 60 | 1.75 | 5.72 ± 12.77 | 26 | 7.69 |
|  | 72 | 1.50 | 2.57 ± 2.68 | 25 | 4.00 |
|  | 84 | 1.20 | 3.98 ± 6.28 | 23 | 13.04 |
|  | 96 | 1.15 | 2.71 ± 3.25 | 20 | 5.00 |
| Treatment^1^ | 0 | 1.60 | 2.56 ± 3.33 | 75 | 4.00 |
|  | 12 | 2.15 | 2.91 ± 3.17 | 57 | 3.51 |
|  | 24 | 2.30 | 5.36 ± 12.72 | 51 | 5.88 |
|  | 36 | 2.50 | 3.89 ± 5.15 | 37 | 5.41 |
|  | 48 | 2.45 | 3.65 ± 5.21 | 33 | 6.06 |
|  | 60 | 2.45 | 3.92 ± 5.38 | 26 | 7.69 |
|  | 72 | 2.80 | 3.43 ± 3.75 | 24 | 4.17 |
|  | 84 | 2.40 | 3.28 ± 3.44 | 18 | 5.56 |
|  | 96 | 2.90 | 2.87 ± 2.13 | 20 | 0.00 |

^1^Data was censored for those participants off-protocol.

^2^Normal Range for CRP in blood is up to 10 mg/L (as per Eastern Ontario Regional Laboratory Association normal reference range).

^3^Percentage (%) Frequency High refers to number of times a reading was more than 10 mg/L normalized to the number (n) of total readings at that time point.
