# Supplementary material for: A randomized control trial of high-dose micronutrient-antioxidant supplementation in healthy persons with untreated HIV infection
Source: PLoS One. 2022 Jul 14;17(7):e0270590. doi: 10.1371/journal.pone.0270590 (PMC9282469; doi:10.1371/journal.pone.0270590)
Supplement: S12 Table — (DOCX) [file pone.0270590.s022.docx]

**SUPPLEMENTAL TABLE 12**  Creatinine measurements (in blood) taken quarterly over the study period in Control (100% recommended daily allowance supplement) and Treatment (High-dose supplement) groups.

|  | Time (Weeks) | Median  (μmol/L) | Mean ± SD  (μmol/L) | n | % Frequency High^2,3^ |
| --- | --- | --- | --- | --- | --- |
| Control^1^ | 0 | 75.0 | 75.64 ± 13.62 | 76 | 6.58 |
|  | 12 | 74.0 | 75.63 ± 13.70 | 60 | 15.00 |
|  | 24 | 73.0 | 74.88 ± 13.76 | 56 | 5.36 |
|  | 36 | 78.0 | 75.43 ± 12.41 | 47 | 4.26 |
|  | 48 | 77.0 | 94.37 ± 110.48 | 41 | 14.63 |
|  | 60 | 78.0 | 75.89 ± 11.40 | 27 | 3.70 |
|  | 72 | 79.0 | 78.41 ± 9.96 | 27 | 7.41 |
|  | 84 | 76.0 | 79.44 ± 15.80 | 25 | 8.00 |
|  | 96 | 77.0 | 76.13 ± 10.39 | 23 | 8.70 |
| Treatment^1^ | 0 | 80.0 | 88.13 ± 87.31 | 83 | 15.66 |
|  | 12 | 77.0 | 76.69 ± 12.24 | 65 | 9.23 |
|  | 24 | 74.0 | 77.19 ± 14.90 | 53 | 11.32 |
|  | 36 | 76.0 | 76.74 ± 14.45 | 43 | 9.30 |
|  | 48 | 75.0 | 76.65 ± 15.64 | 37 | 13.51 |
|  | 60 | 76.5 | 77.19 ± 14.13 | 32 | 12.50 |
|  | 72 | 78.0 | 76.57 ± 16.59 | 21 | 9.52 |
|  | 84 | 75.0 | 77.85 ± 17.20 | 20 | 10.00 |
|  | 96 | 75.0 | 79.74 ± 27.41 | 19 | 15.79 |

^1^Data was censored for those participants off-protocol.

^2^Normal Range for Creatinine in blood is 22-75 μmol/L (females) and 49-93 μmol/L (males) (as per Eastern Ontario Regional Laboratory Association normal reference range).

^3^Percentage (%) Frequency High refers to number of times a reading was more than 93 μmol/L normalized to the number (n) of total readings at that time point.
