# Supplementary material for: A randomized control trial of high-dose micronutrient-antioxidant supplementation in healthy persons with untreated HIV infection
Source: PLoS One. 2022 Jul 14;17(7):e0270590. doi: 10.1371/journal.pone.0270590 (PMC9282469; doi:10.1371/journal.pone.0270590)
Supplement: S13 Table — (DOCX) [file pone.0270590.s023.docx]

**SUPPLEMENTAL TABLE 13** Total Protein measurements (in blood) taken quarterly over the study period in Control (100% recommended daily allowance supplement) and Treatment (High-dose supplement) groups.

|  | Time (Weeks) | Median  (g/L) | Mean^2^ ± SD  (g/L) | n |
| --- | --- | --- | --- | --- |
| Control^1^ | 0 | 78.0 | 77.97 ± 6.75 | 76 |
|  | 12 | 78.0 | 78.44 ± 6.26 | 59 |
|  | 24 | 79.0 | 79.38 ± 6.12 | 55 |
|  | 36 | 79.0 | 79.74 ± 6.16 | 46 |
|  | 48 | 80.0 | 80.53 ± 6.36 | 40 |
|  | 60 | 76.0 | 78.42 ± 7.40 | 26 |
|  | 72 | 79.0 | 78.46 ± 6.22 | 26 |
|  | 84 | 78.0 | 77.92 ± 5.52 | 25 |
|  | 96 | 78.0 | 79.38 ± 7.53 | 21 |
| Treatment^1^ | 0 | 78.0 | 78.77 ± 7.48 | 83 |
|  | 12 | 78.0 | 78.86 ± 7.32 | 63 |
|  | 24 | 78.0 | 77.55 ± 9.32 | 49 |
|  | 36 | 78.0 | 78.00 ± 6.46 | 40 |
|  | 48 | 78.0 | 78.73 ± 6.29 | 37 |
|  | 60 | 80.0 | 78.76 ± 6.43 | 29 |
|  | 72 | 77.0 | 77.77 ± 6.91 | 22 |
|  | 84 | 79.0 | 78.84 ± 5.78 | 19 |
|  | 96 | 79.0 | 79.63 ± 5.42 | 19 |

^1^Data was censored for those participants off-protocol.

^2^Normal Range for total protein in blood is up to 64-82 g/L (as per Eastern Ontario Regional Laboratory Association normal reference range) but we have not quantified the high readings.
