# Supplementary material for: A randomized control trial of high-dose micronutrient-antioxidant supplementation in healthy persons with untreated HIV infection
Source: PLoS One. 2022 Jul 14;17(7):e0270590. doi: 10.1371/journal.pone.0270590 (PMC9282469; doi:10.1371/journal.pone.0270590)
Supplement: S1 File — (DOC) [file pone.0270590.s024.doc]

**Research Ethics Board**

**Conseil d'éthique en recherches**

**798-5555 ext 14146, 14902 or 15072**

**Fax No. ~ 761-4311**

[**http://www.ohri.ca/ohreb/**](http://www.ohri.ca/ohreb/)

#

# **Application for Research Ethics Approval**

To submit a research proposal to the Ottawa Hospital Research Ethics Board (OHREB), it is mandatory to submit the actual protocol.

The application forms must be filled out completely. Please bear in mind that some members of the OHREB may not be experts in your own field so please strive, where appropriate, to make your submission understandable to those outside your area of expertise. Information Sheets and Consent Form must be included with the protocol (where appropriate).

Please submit the appropriate number of copies:

For protocols requiring **full review**:

- Application 18 copies (signed original plus 17 copies)
- Patient Information Sheet and/or

Consent forms * 18 copies (original plus 17 copies)

(* English version only)

(Once the English version has been approved by the OHREB, the French version may be submitted.)

- Departmental Impact Sheet

with applicable attachments 18 copies (signed original plus 17 copies)

- Detailed Budget: 18 copies
- Protocol: 8 copies
- Questionnaires,

Case Report Forms, etc : 8 copies

- Investigator’s Brochure: 2 copies

For protocols requiring **expedited review :**

**(Survey, Chart Reviews, Non-Invasive Testing/Intervention):**

- Application: 2 copies (signed original plus 1 copy)
- Patient Information Sheet and/or

Consent forms * 2 copies (original plus 1 copy)

- Questionnaires, etc.

(* English version only)

(Once the English version has been approved by the OHREB, the French version may be submitted.)

- Departmental Impact Sheet with

applicable attachments 2 copies (signed original plus 1 copy)

- Detailed Budget 2 copies (original plus 1 copy)
- Protocol 2 copies (original plus 1 copy)

N.B. All documentation must be typed. Incomplete protocols will be returned prior to review.

***TIPS***

The forms have been created in Word, using the Forms tool to allow ease of use in completing them. The following are a few tips on completing the document:

- Please use the “TAB” key to move between fields.
- The “Enter” key will create extra space in the form that is not required.
- The form fields will not accept formatting, normal type will be fine.
- It is hoped that you are able to complete the form in the space allocated, however, if you need additional space, please insert a page where appropriate.
- If you have suggestions to improve this form, please let us know.

## **Protocol Submission**

The application form, the protocol, investigator’s brochure, the consent form, patient information sheet, budget, questionnaires and any other material described above should be submitted to:

Ottawa Hospital Research Ethics Board
c/o Ms. Christine Banyard
Ottawa Hospital, Civic Campus

751 Parkdale Avenue

Suite 106

Ottawa, Ontario K1Y 1J7

- or -

Ottawa Hospital Research Ethics Board
Ottawa Hospital Research Institute – General Campus
Cancer Clinic Building
501 Smyth Road
4^th^ Floor
Ottawa, Ontario K1H 8L6

**To confirm whether or not we have received your correspondence, or to request a copy of lost correspondence, please contact:**

Ida Ianni-Lucio

Ethics Clerk

798-5555, extension 13523

**For information on the status of your protocol up to and including initial approval, please contact:**

Christine Banyard
Secretary, Research Ethics Board
798-5555, extension 14902

**For general inquiries about research ethics including the status of revised information sheets, amendments, renewals, terminations, etc., please call:**

Patricia Sorgat
Protocol Officer, Research Ethics Board
798-5555, extension 14146

**For information on procedural issues, problematic protocols, compensation clauses, OHREB document changes, etc. please contact:**

Mary Ann Laviolette

Ethics Co-ordinator

798-5555, extension 15072

**For information on and requests for clinical research space, please contact:**

Janet Whyte

Clinical Research Manager

798-5555, ext. 12681

jwhyte@ohri.ca

**The Chair of the OHREB is:**

Raphael Saginur, M.D.
798-5555, extension 14902

Protocols must be signed by the Head of the Department or Division in which the research projects are to be carried out. (The Principal Investigator or Co-Investigator should not sign as the Head of the Department or Division for their own research studies)

Protocols will be reviewed on a bi-monthly basis at the OHREB meeting. Please refer to the list for the exact submission deadlines listed under ‘Meeting Dates’.

Protocols submitted for ‘Expedited Review’ are normally processed within two to four weeks of submission. Applications may be submitted at any time – the submission dates do not apply to ‘expedited’ studies.

Protocols submitted for ‘Chart Review’ are normally processed within 24 to 48 hours of submission. Applications may be submitted at any time – the submission dates do not apply to ‘chart review’ studies.

(Revised August 5, 2005)

# **RESEARCH ETHICS APPLICATION**

## **NOTE:** A**ll documentation must be type written.**

Use the “TAB” key to move between fields.

1. **PROTOCOL TITLE**

| A RANDOMIZED CONTROL CLINICAL TRIAL OF MICRONUTRIENT & ANTIOXIDANT SUPPLEMENTATION IN PERSONS WITH UNTREATED HIV INFECTION. The MAINTAIN Study |
| --- |

1. **PRIMARY INVESTIGATOR FOR MULTICENTRE TRIALS**

| Last Name | Cameron | First Name | Bill |
| --- | --- | --- | --- |
| Title/Position | Professor | Department  & Location | Medicine |
| Tel. | 613-737-8923 |
| Email | bcameron@ohri.ca | Fax | 613-737-8925 |

1. **PRINCIPAL INVESTIGATOR AT THE OTTAWA HOSPITAL**

*If the Principal Investigator does not hold an academic appointment, indicate who the responsible investigator will be.*

| Last Name | Cameron | First Name | Bill |
| --- | --- | --- | --- |
| Title/Position | Professor | Tel. | (613)737-8923ext. |
|  |  | Fax | (613)737-8925 |
| Dept/Unit & Location | Medicine | Email | bcameron@ohri.ca |
| Division/  Portfolio | Infectious Diseases | Signature: |  |

1. **CO-INVESTIGATORS AT THE OTTAWA HOSPITAL**

| Last Name | Angel | First Name | Jonathan |
| --- | --- | --- | --- |
| Title/Position | Associate Professor | Tel. | (613)737-8442 ext. |
|  |  | Fax | (613)737-8164 |
| Dept/Unit & Location | Medicine | Email | jangel@ohri.ca |
| Division/  Portfolio | Infectious Diseases | Signature: |  |

| Last Name | Garber | First Name | Gary |
| --- | --- | --- | --- |
| Title/Position | Professor and Head | Tel. | (613)737-8173 ext. |
|  |  | Fax | (613)737-8099 |
| Dept/Unit & Location | Medicine | Email | ggarber@ottawahospital.on.ca |
| Division/  Portfolio | Infectious Diseases | Signature: |  |

| Last Name | Cooper | First Name | Cutis |
| --- | --- | --- | --- |
| Title/Position | Associate Professor | Tel. | (613)737-8924 ext. |
|  |  | Fax | (613)737-8164 |
| Dept/Unit & Location | Medicine | Email | ccooper@ottawahospital.on.ca |
| Division/  Portfolio | Infectious Diseases | Signature: |  |

| Last Name | Lee | First Name | Craig |
| --- | --- | --- | --- |
| Title/Position | Associate Professor | Tel. | (613)737-8051 ext. |
|  |  | Fax | (613)737-8164 |
| Dept/Unit & Location | Medicine | Email | clee@ottawahospital.on.ca |
| Division/  Portfolio | Infectious diseases | Signature: |  |

| Last Name | MacPherson | First Name | Paul |
| --- | --- | --- | --- |
| Title/Position | Assistant Professor and Staff Scientist | Tel. | (613)737-8899 ext. 73917 |
|  |  | Fax | (613)737-8164 |
| Dept/Unit & Location | Medicine | Email | pmacpherson@ottawahospital.on.ca |
| Division/  Portfolio | Infectious Diseases | Signature: |  |

***If you need more space an additional form for co-investigators is available on the website.***

1. **APPROVAL BY INVESTIGATOR’S DEPARTMENT/DIVISION HEAD/**

CLINICAL MANAGER/CHIEF (This should not be the Principal Investigator and/or Co-Investigator)

| Hospital and university division and department administrators are responsible for academic activities within their unit, and may provide guidance and support to investigators. The purpose of this signature section is to ensure that administrators are aware of research activities and the impact of these activities.  □ The study answers a reasonable scientific/clinical question and is consistent with  hospital/faculty policies and mission.  □ The study resources (budget, space, support staff) are adequate to support the  study.  □ The local investigators are qualified to perform the study.  □ There are an adequate number of research participants suitable to be  approached for enrolment for this study. This population is not already over-  subscribed in clinical research.  **I have reviewed this application and agree it should be submitted for ethics approval.** | | |
| --- | --- | --- |
| Name | Dr. Jeff Turnbull | Contact Number : (613)737-8900 ext. |
| Title/Position | Chief of Medicine | Signature |
| Dept/Unit & Location | Medicine |
| Date |  |

**6. REVIEW TYPE**

Please indicate whether you are requesting full or expedited review. (Please see our website [www.ohri.ca/ohreb](http://www.ohri.ca/ohreb) for more information on what qualifies for expedited review.)

|  | Full Review |
| --- | --- |
|  | Expedited Review |

7. STUDY TYPE

| Describe the Research Project by checking as many of the following as apply:  Study Type: | | | | | | | | |
| --- | --- | --- | --- | --- | --- | --- | --- | --- |
|  | Investigator driven and sponsored by OHRI | | | | | | | |
|  | Experimental Research/Clinical Trial | | | | | | | |
|  | Observational Research | | | | | | | |
|  | Pilot | | | | | | | |
|  | Sequel to previously approved project (Protocol #:      ) | | | | | | | |
|  | Genetic Research (Addendum 1 must be included with completed application) | | | | | | | |
|  | Program Evaluation | | | | | | | |
|  | New therapeutic method | | | | | | | |
|  | Medical Device Research  Please attach a letter from sponsor indicating Health Canada application/approval.  This is mandatory prior to final REB approval. | | | | | | | |
|  |  | Health Canada Application/Approval is attached (insert as next page) | | | | | | |
|  |  | Health Canada Application/Approval will be forwarded. | | | | | | |
| Location of the Study: | | | | | | | | |
|  | Single Centre Trial | | | |  | Multicentre Trial | | |
| Study Design: | | | | | | | | |
|  | Randomized, controlled trial | | | | | | | |
|  | Phase I | |  | Phase II |  | Phase III |  | Phase IV |
|  | Open Label | |  | Single Blinded |  | Double Blinded (or more) | | |
|  | Case-Control study | | | | | | | |
|  | Cohort study | | | | | | | |
|  | Interview, survey or questionnaire, observation | | | | | | | |
|  | Chart Review | | | | | | | |
|  | Compassionate Use | | | | | | | |
|  | N of 1 Study | | | | | | | |
|  | Quality Assurance | | | | | | | |
| **Drug Study:**  **Yes**  **No** | | | | | | | | |
|  | Involves unusually elevated doses of available drugs | | | | | | | |
|  | Involves anticipated high incidence of toxicity | | | | | | | |
|  | Drug being used for currently approved indication | | | | | | | |
|  | Approved Drug being used for a non-approved indication  Please attach a letter from sponsor indicating Health Canada application/approval.  This is mandatory prior to final REB approval. | | | | | | | |
|  |  | Health Canada Application/Approval is attached (insert as next page) | | | | | | |
|  |  | Health Canada Application/Approval will be forwarded. | | | | | | |
|  | Investigational non-approved drug:  Please attach a letter from sponsor indicating Health Canada application/approval.  This is mandatory prior to final REB approval. | | | | | | | |
|  |  | Health Canada Application/Approval is attached (insert as next page) | | | | | | |
|  |  | Health Canada Application/Approval will be forwarded. | | | | | | |

###### 8. PURPOSE AND OBJECTIVES

| Please state clearly the hypothesis to be tested, in lay terms.  **HYPOTHESES**  **Primary:** Supplementation of untreated HIV positive persons with broad spectrum micronutrients and antioxidants will delay decline in CD4 T lymphocyte count <350 cells/mm^3^ or start of ART compared with multivitamins.  **Secondary:** Supplementation of untreated HIV positive persons with broad spectrum micronutrients and antioxidants will be well tolerated and safe.   - 1. **Primary Objective** - To evaluate if supplementation with study medication will delay decline of absolute CD4+ cell count or start of ART versus control supplement   1. **Secondary Objectives** - To evaluate all adverse events - To evaluate if study medication is well tolerated - To evaluate if study medication can delay initiation of ART - To evaluate effect of study medication on immune cells - To evaluate effect of study medication on laboratory tests related to safety - To evaluate effect of study medication on plasma HIV RNA viral load - To evaluate effect of study medication on AIDS related event - To evaluate effect of study medication on quality of life - To evaluate dietary intake |
| --- |

| Provide the rationale for the study.  Micronutrient deficiencies occur even in early stages of human immunodeficiency virus (HIV) infection. Micronutrient deficiencies increase risk of disease progression to acquired immunodeficiency syndrome (AIDS) and morbidity and mortality, but the role of micronutrient antioxidant supplements in medical management of HIV/AIDS is not clear. |
| --- |

| **STUDY DURATION** | Start Date:**/01/2008**  DD/MM/YYYY | End Date:**/ /2011**  DD/MM/YYYY |
| --- | --- | --- |

###### 9. RECRUITMENT

| Describe how these research participants will be identified and recruited. **(Only members of the participant’s health care team should contact patients at the Ottawa Hospital.)**  In addition, please ensure you address the following:   - If initial contact is by letter or if an **advertisement** is to be used, attach a copy. - How will the researcher ensure that there are no breaches of patient **privacy**? - How will the possibility of **coercion**, duress or undue incentive be avoided or minimized?   Participants will be approached by the investigators and study staff during routine patient clinic visits. The investigator (regular treating physician at the clinic) will make initial contact with a possible study participant, informing them of their possible eligibility for the study. If the possible participant seems interested in hearing more about the study, the investigator will inform them that a study coordinator is available to discuss the study in further detail, including their role in participating.  The investigator then leaves the room and the coordinator enters. She will explain the study in detail, answer all questions, and review the informed consent which includes all possible risks associated with participating. The possible participant is told they may take the consent form home to reread, and discuss with friends/family about participating.  The coordinator along with the investigator are sure to inform the possible participant that if they decide that they do not wish to participate, that there will be no impact to the regular care which they receive at the clinic.  Privacy will be ensured by utilizing a 4-digit study subject identification code to correspond to treatment data. As mandated by the hospital, patients' medical records will not leave the hospital. Records and related information will be kept confidential. Patient initials will be used only by the study coordinator. Any forms which contain the patient’s initials will not leave the hospital.  Advertisement will be used for this study. Once the advertisement is approved, it will be posted in the treatment rooms in Module G-12 (Infectious Disease Clinic), at the General Campus. There are 7 treatment rooms. Only patients, nurses and physicians have access to these treatment rooms. In addition, the advertisement will be posted in various private clinics across Ottawa. |
| --- |

| Are recruitment incentives provided?  Yes  No  If yes, please describe.  Participants will be provided with out-of-pocket expenses (~ $20.00/visit). |
| --- |

| Are controls involved?  Yes  No  Does the study include subjects in a control group?  Yes  No  If controls are involved, if their selection and/or recruitment differs from the above, provide details.    Same recruitment |
| --- |

| Does the research include research participants who may not be competent to give informed consent?  Yes  No If yes, justify and explain how consent will be obtained and from whom. | | | |
| --- | --- | --- | --- |
| - Number of Centres recruiting globally: **20-22 Sites across Canada**   National Study – Country **Canada**  International Study – Country | | | |
| - Total number of research participants being recruited at all centres globally: **210** | | | |
| - Number of research participants to be recruited at each Ottawa Hospital campus: | | | |
| Civic | General  **50** | Riverside | Other |
| Provide the rationale for the selected sample size and the methodology used to calculate the sample size:  Estimated time to CD4 count <350 cells/mm^3^ or start of ART was based on previous research^49^ and expert opinion. Based on the UK study, we estimated that the control median time from baseline to CD4 count <350 cells/mm^3^ or start of ART to be 50% at 2 years. We hypothesized that treatment with micronutrients and antioxidants would decrease the median to 32% (18% absolute change in median at 2 years). A two-sided log rank test with an overall sample size of 210 subjects (of which 105 are in the treatment group and 105 in the control group) achieves 80% power at a 0.05 significance level to detect a difference of 18% between the proportions surviving in the control and treatment groups after 2 years. Patients will be enrolled during an accrual period of one year and 50% of the enrollment will be complete when 50% of the accrual time has past. We have also accounted for a modest non-compliance rate of approximately 10% over the two years for those participants allocated to active treatment medication and 15% for those allocated to control. See Appendix A of the protocol for detailed calculation. | | | |

### 10. DESCRIPTION OF POPULATION

| **Inclusion Criteria** - Who is being recruited and what are the criteria for their selection?  The participant must:   - be HIV infected adult, at least 18 years of age - have CD4+ cells ≥350 and ≤800 cells/mm^3^ - have HIV RNA level <100,000 copies per mL - have received no ART (excluding less than seven days and prenatal transmission prophylaxis) - if a woman of child bearing potential, have a negative pregnancy test within two weeks prior to randomization and agree to practice barrier method of birth control during the study - be willing and able to sign informed consent and to comply with the study protocol |
| --- |

| **Exclusion Criteria** - What research participants are excluded from participation?  The participant must not:   - have known allergy or intolerance to any study medication ingredient - be pregnant - have active treatment for an acute opportunistic infection or malignancy - have ALT greater than 5X normal range - have serum creatinine >133 μmol/L - abuse alcohol and recreational drugs - taking micronutrient (except vitamin D) or herbal supplements within 30 days of randomization |
| --- |

11. DESCRIPTION OF METHODS AND PROCEDURES

If additional space is required, insert one additional page)

| **Summary of Methods and Procedures.** **Please include a summary of the following**:   - any specific manipulations; - type, quantity, and route of administration of drugs and radiation; operations; tests; use of medical devices that are prototype or altered from those in clinical use; - type, and number of interviews or questionnaires; are the questionnaires validated, reliable and have they been pilot tested? - Are procedures/treatments standard practice or new, what is the risk level?   **(Flow diagrams and point form discussion are encouraged and should be appended separately.)**  **Study design:** A prospective, randomized, controlled, double blind clinical trial of supplementation of 210 untreated HIV infected adults with a micronutrient and antioxidant preparation or identical appearing RDA multivitamins and minerals for two years with quarterly follow up in clinic for assessment of time from baseline to CD4 count <350 mm^3^ or start of ART.  **STUDY FLOWCHART**  210 consenting eligible participants will be enrolled in routine clinic visits and randomized to two groups  Neither research staff nor participants will know assigned treatment  Treatment group  n=105  Will receive daily a micronutrient and antioxidant preparation for two years  Control group  n=105  Will receive identical appearing multivitamins and minerals  Participants will be followed in quarterly clinic visits for two years  They will be offered opportunistic infection prophylaxis and begin ART as indicated according to current treatment guidelines  On reaching primary outcome (CD4+ cells <350/mm^3^ or start of ART), participants in both groups will be offered open label study medication with continued follow up until end of study, but censored from analysis  Analyses of primary outcome by intention-to-treat of all participants randomized will compare time from baseline to CD4 count <350 cells/mm^3^ or start of ART between groups  Secondary outcomes relate to indicators of safety, well being and health  **Table 1: Study Schedule**   |  | **Screen Weeks –4 to -2** | **Baseline Week 0** | **Weeks 12, 24, 36, 48, 60, 72, 84, and 96 Weeks**  **and Study Termination Visit*** | | --- | --- | --- | --- | | Informed consent | x |  |  | | Medical history** | x |  |  | | Physical examination | x |  |  | | Subjective complaints |  |  | x | | Objective findings*** |  |  | x | | Adverse events |  |  | x | | AIDS related illness |  |  | x | | Resolution of AIDS related illness |  |  | x (if applicable) | | Height and weight |  | x | x | | Medications taken† | x | x | x | | Blood chemistry†† | x | x | x | | Hematology‡ | x | x | x | | Coagulation tests (INR) |  | x | x | | Plasma HIV RNA | x | x | x | | Lymphocyte measures‡‡ | x | x | x | | Hepatitis serology¶ | x |  |  | | Quality of life questionnaires║ |  | x | x | | Collect optional 7-day food diary (given at previous visit to consenting participants) |  | x | x  (Weeks 24, 48, 72 and 96) | | Study medications pill count |  |  | x | | Dispense study medications |  | x | x  (up to Week 84) | | Urinalysis | x | x | x | | Pregnancy test§ | x |  |  |   Tests are acceptable if results of testing within one month of visit are available  *Study termination visit will be 2-6 weeks after Week 96 visit or after premature withdrawal from the study  **Medical history including previously diagnosed medical conditions, previous opportunistic infections, and previous and current medications, including ART, alcohol, illicit and recreational drug, and herbal preparation use  ***Weight change, fever, diarrhea, oral hairy leukoplakia, lymphadenopathy, hepatosplenomegaly  †Other medications including ART, alcohol, illicit and recreational drug, and herbal preparation use  ††Fasting glucose, blood urea nitrogen (BUN), Hgb A1C, creatinine, total protein, albumin, alkaline phosphatase, lactate, alanine transaminase (ALT), aspartate transaminase (AST), amylase, lipase, total bilirubin, fasting cholesterol, triglycerides, beta-carotene, vitamins B_6_ and D (25-OHD) and folate.  ‡White blood cell (WBC) count with differential, red blood cell count (RBC), hemoglobin, hematocrit, platelet count  ‡‡ALC, CD4+, CD3+, and CD8+ cell counts, CD4%, CD8%, CD4:CD8 ratio. Each participant will have two measures of CD4+ cells at least one and up to six weeks apart to estimate mean baseline and end-of-study values (96 weeks)  ¶(HBV DNA or HBsAg) and (HCV RNA or HCV Ab) if not already documented  ║MOS-HIV, EuroQol, and Health Utilities Index (HUI)  §If urine pregnancy test is positive, a serum β-HCG test will be performed. After the screening visit, a pregnancy test will be done only as needed (i.e., if menses are delayed) |
| --- | --- | --- | --- | --- | --- | --- | --- | --- | --- | --- | --- | --- | --- | --- | --- | --- | --- | --- | --- | --- | --- | --- | --- | --- | --- | --- | --- | --- | --- | --- | --- | --- | --- | --- | --- | --- | --- | --- | --- | --- | --- | --- | --- | --- | --- | --- | --- | --- | --- | --- | --- | --- | --- | --- | --- | --- | --- | --- | --- | --- | --- | --- | --- | --- | --- | --- | --- | --- | --- | --- | --- | --- | --- | --- | --- | --- | --- | --- | --- | --- | --- | --- | --- | --- | --- | --- | --- | --- | --- | --- | --- | --- |

### 12. RISKS

| Describe the discomfort or risks that participants may incur as a result of their participation in this research. Also note the following:particular risks associated with each procedure, drug, test or other aspect of the protocol.delineate, when appropriate, what risks relate to standard care,what risks relate to participation in the study.quantify risks where possible by providing percentages, or by describing as rare, common, etc. *Safety of the proposed supplement:* The proposed supplement has been used safely for three months in HIV-infected individuals ^21^ and is available over the counter in the United States. Other longer studies of micronutrient and antioxidant supplementation in comparable dosage in HIV-infected and -uninfected adults show no harm ^38,42^. A meta-analysis of clinical trials shows no harm of antioxidant supplementation, except possible increased mortality with some antioxidants in a secondary analysis ^43^. A possible adverse effect of β-carotene in smokers and those exposed to asbestos ^44^ and with high intake in HIV infected adults in an observational study ^45^ have not been confirmed in large randomized studies. Carotenemia and skin discoloration (both reversible) have not been reported in HIV infection even with beta-carotene doses higher than proposed here. Supplementation of nucleoside reverse transcriptase inhibitor-treated HIV-infected adults with vitamins C and E and NAC was associated with increased fasting glucose levels at 24 weeks, but this study was small and uncontrolled and all the participants either had lipoatrophy or sustained hyperlactatemia ^46^. These and other micronutrients and antioxidants were well tolerated in other studies ^21,38^. There is no evidence of harm of iron supplementation in HCV co-infected HIV infected adults ^47^, and micronutrient and iron supplementation of coinfected female injection drug users improves anemia without increasing plasma HCV or HIV RNA levels or altering liver enzymes ^48^, and no harm has ever been shown at the proposed iron dosage of 18 mg. Hepatitis coinfection patients are more likely to be nutrient depleted and oxidative stressed, which seem to be related to effectiveness of the supplement. |
| --- |

| Will the management of the participant’s condition be prolonged or delayed as a result of the research?  Yes  NoIf yes, explain, specifying any risks associated with prolongation or delay. |
| --- |

| Are there any standard therapies, diagnostic procedures or information to be withheld from participants for the purpose of the study?  Yes  No  If yes, explain, specifying the risks and benefits to the participants and justify. |
| --- |

| Are there stopping rules for the study? **(This does not apply to individual patient withdrawal, but to the study as a whole)**   Yes  No  If yes, please describe. |
| --- |

| Is there a data safety monitoring board in place?  Yes  No  If yes, describe the composition of the board.  Adverse events attributable to the intervention and laboratory parameters of safety will be assessed and monitored by the Data Safety and Monitoring Board (DSMB). If there is concern for safety as a result of the study, or due to new information regarding the study formulation or its components, the Study Steering Committee will make a decision regarding dissemination of the information to the participants. If new information regarding the study or the study medication or its components raises concerns for the safety of the participants, the Study Steering Committee will defer the decision to CTN’s Safety and Efficacy Review Committee (SERC), an autonomous body with authority to discontinue the study. The DSMB and all study committees will include a community representative. |
| --- |

| What procedures in this protocol are additional to those required for **routine** patient care? **(Include the Impact Sheet with applicable attachments for all procedures which are attributable to this study)**  None |
| --- |

| Is a placebo used?  Yes  No  If yes, please justify:  Placebo is a RDA vitamin and mineral preparation that is identical in appearance to the oral broad spectrum micronutrient and antioxidant preparation being studied. |
| --- |

### 13. POSSIBLE BENEFITS

| Describe any possible benefits to the participant as a result of their participation in this research. Patients are not guaranteed any direct benefit from participating. The knowledge gained from this study may be helpful to others in the future. |
| --- |

###### 14. CONFIDENTIALITY

| How will data be protected against breaches in security/privacy? If the information will be housed in a database, will it be password protected? There should not be any patient identifiers such as hospital unique number, OHIP #, etc. (An independent study number should be assigned to each patient, and the link between the patient and the study number housed separately and securely) Will there be written documentation kept? If so, how long will the information be kept? Where? Who will have access? **It is the policy of the OHREB that no records with the patient’s name leave the Ottawa Hospital. For all drug studies, records must be kept by the investigator for a period of 25 years as outlined in Health Canada’s Food and Drug Regulations, Division 5 – Drugs for Clinical Trials Involving Human Subjects, Section C.05.012. For all other studies, records must be kept by the investigator for a period of 15 years.**  No records bearing the participant's name will leave the hospital. Participants will be identified only by a 4-digit study identification number. The information will be kept in the hospital in a locked filing cabinet and locked room. The link between the patient’s name and code will be stored in the Study Coordinator’s office in a binder that only she and her research assistant will have access to. All REB Approved copies of the Patient Informed Consent Forms will be stored in the study’s Regulatory binder. Once a consent form has been printed for submission to the REB for approval, the electronic version is then password protected so that it may not be altered in any way between the time it is submitted until approval and then from approval to renewal or amendment if applicable. The password is kept confidential in the office of the research assitant in charge of regulatory documentation. The study doctor and his staff, the Canadian HIV Trials Network (CTN), Health Canada and other regulatory agencies such as The Ottawa Hospital Research Ethics Board will have access to the information, but will be bound by the confidentiality guidelines of The Ottawa Hospital. Publications or presentations will not identify participants by name. Once the study is complete, study documents will be stored at the hospital in a locked room for as long as the space is available. Once the storage space runs out, the boxes will be transferred to a long term storage facility (Securit) for the remainder of the 25 year storage period. The transfer of the study information will be documented. At the end of the 25 year storage period, documents will be shredded to ensure patient confidentiality. All electronic data and documentation will also be deleted at the end of the 25 year storage period, and its destruction will be documented. |
| --- |

### 15. BUDGET

| Has this research been funded?  **YES**  **NO** | | |
| --- | --- | --- |
| **If YES provide the:**  Name of the Agency/Sponsor:  Amount of funding received: | If NO complete the following:  Has funding been applied for:  **YES**  **NO**  Name of the Agency/Sponsor: **OHTN and CTN**  Amount of funding applied for**: $ $150,000 (first yr.)**  **(~$125,000 in 2^nd^ and 3^rd^ years respectively.**  Date submitted:  **1/11/2007**  DD/MM/YYYY | |
| If no funding has been applied for, how will the research be supported?  If the study was peer-reviewed, please provide the name of the individual or agency that completed the review. | | |
| Does the industry-sponsored budget contain appropriate overhead?  A Clinical Trial Agreement has been forwarded to the Contracts  Officer  A Clinical Trial Agreement is attached to the OHREB  application  Have you included a budget summary for the protocol?  **(If this is not included, approval of protocol will be delayed.**  **Details on the disbursement of any excess funds received for this study should also be provided.)** | | **YES**  **NO**  **N/A**  **YES**  **NO**  **N/A** |
| Does the REB review fee apply to this study?  A cheque is enclosed  An invoice request is enclosed  A transfer request is enclosed  **(All industry sponsored research projects are subject to a $2,000.00 administration fee, and any cooperative group studies that are funded in excess of $3000 per patient.)** | | **YES**  **NO**  **N/A** |
|  | |  |
| **POTENTIAL CONFLICTS OF INTEREST**  Please indicate if you have a conflict of interest or separate financial agreements with the sponsor of this study.  If yes, please explain. | | **YES**  **NO**  **N/A** |

### 16. STAFFING

| What staffing will be required? All staff should be listed and this list maintained during the course of the study.  This includes research nurses, research co-ordinators, etc. Research nurses should provide a copy of their Nursing Certificate for the current year, to the Research Services Office if this has not already been done for another study. | | | |
| --- | --- | --- | --- |
| **Name &**  **Role in Study** | **Already Employed?** | **Full-time vs.**  **Part-time** | **Office/Laboratory Location and Telephone Number**  **If new office or laboratory space is required, the Space Planning & Management section of the Departmental Impact form must be signed and submitted** |
| 1. Nancy Lamoureux RN  Study Coordinator | **YES**  **NO** | **FT**  **PT** | **Campus:** General  **Office/Lab # K15-B**  (613) 737-8209 **x.** |
| 2. Linda Longpre  Regulatory Coordinator | **YES**  **NO** | **FT**  **PT** | **Campus:** General  **Office/Lab # 108(portable)**  (613) 737-8899 **x.74706** |
| 3. Mary-Ellen Arsenault  Research Assistant | **YES**  **NO** | **FT**  **PT** | **Campus:** General  **Office/Lab # K15-B**  (613) 737-8879 **x.** |
| 4. | **YES**  **NO** | **FT**  **PT** | **Campus:**  **Office/Lab #** |
| 5. | **YES**  **NO** | **FT**  **PT** | **Campus:**  **Office/Lab #**  (   )    -     **x.** |
| In the space below please provide: | | | |
| 1. a list of their qualifications: Nancy Lamoureux is a Research Coordinator and RN (her license is on file with the REB), Linda Longpré is a Reseach Assistant responsible for regulatory documents, Mary-Ellen Arsenault is a Research Assistant to Nancy Lamoureux responsible for filing, and preperation of patient visits. | | | |
| a list of the duties to be performed, including delegated/sanctioned medical acts and acts performed under the Regulated Health Professions Act if this has not already been done for another study:  Duties performed will be regulatory approval, informed consent, medical history, physical examination including vital signs, Pharmokinetic testing, case report forms and record keeping, collection of blood, urine, and spinning and preparation of blood samples into aliquots (where needed). | | | |

**Research Ethics Board**

**Conseil d'éthique en recherches**

**798-5555 ext 14146, 14902 or 15072**

**Fax No. ~ 761-4311**

[**http://www.ohri.ca/ohreb/**](http://www.ohri.ca/ohreb/)

### DEPARTMENTAL IMPACT

**Protocol Title:**

| A RANDOMIZED CONTROL CLINICAL TRIAL OF MICRONUTRIENT & ANTIOXIDANT SUPPLEMENTATION IN PERSONS WITH UNTREATED HIV INFECTION. The MAINTAIN Study | | | |
| --- | --- | --- | --- |
|  | **Does the protocol require use Hospital and/or OHRI resources (equipment, staff, space) over and above those normally required in the standard care of a patient?** | | |
|  | Equipment | **YES**  **NO** |  |
|  | Staff | **YES**  **NO** |  |
|  | Space | **YES**  **NO** |  |
|  |  | | |
|  | **Will hospitalization or outpatient visits be required beyond what is required for standard care?** | | |
|  | Outpatient Visits | **YES**  **NO** |  |
|  | Hospitalization | **YES**  **NO** |  |
|  |  | | |
|  | **Indicate impacts associated with this Protocol, by Department:**  ***IF YES is indicated, a signature of an individual authorized to sign for the department must be obtained. (Please see our website for a list of contact names*** [http://www.ohri.ca/ohreb](http://www.ohri.ca/ohreb/)***/)*** | | |
|  |  |  | **Signature:** |
|  | Nursing | **YES**  **NO** |  |
|  | Emergency Department | **YES**  **NO** |  |
|  | Health Records  (See Appendix A) | **YES**  **NO** | Please submit signed Health Records form. [www.ohri.ca/ohreb/forms.htm](http://www.ohri.ca/ohreb/forms.htm) |
|  | Laboratory Services | **YES**  **NO** | Please submit signed Lab Impact form. [www.ohri.ca/ohreb/forms.htm](http://www.ohri.ca/ohreb/forms.htm) |
|  | Radioisotopes  (See Appendix B) | **YES**  **NO** | Please submit Radiation Safety form.  [www.ohri.ca/ohreb/forms.htm](http://www.ohri.ca/ohreb/forms.htm) |
|  | Diagnostic Imaging  (See Appendix B and C) | **YES**  **NO** | Please submit signed Diagnostic Imaging form. [www.ohri.ca/ohreb/forms.htm](http://www.ohri.ca/ohreb/forms.htm) |
|  | Pharmacy | **YES**  **NO** | Please submit signed Pharmacy form  [www.ohri.ca/ohreb/forms.htm](http://www.ohri.ca/ohreb/forms.htm) |
|  | Nutrition And Food Services | **YES**  **NO** |  |
|  | Space Planning & Management | **YES**  **NO** |  |
|  | Clinical Investigation Unit | **YES**  **NO** |  |
|  | Cardiopulmonary Services | **YES**  **NO** |  |
|  | Others: | **YES**  **NO** |  |
