# Supplementary material for: A randomized control trial of high-dose micronutrient-antioxidant supplementation in healthy persons with untreated HIV infection
Source: PLoS One. 2022 Jul 14;17(7):e0270590. doi: 10.1371/journal.pone.0270590 (PMC9282469; doi:10.1371/journal.pone.0270590)
Supplement: S2 File — (DOCX) [file pone.0270590.s025.docx]

**Supplementary Methods**

**Statistical Analyses of Secondary Outcomes**

The participants were followed from baseline out to 96 weeks for the secondary outcome measures. Secondary outcome measures include: CD4 T lymphocyte counts, CD8 T lymphocyte counts, CD4:CD8 ratio, HIV viral load and serum chemistry (detailed below).

CD4 T lymphocyte count (cells/μL) and HIV viral load (copies/mL) measures were censored two ways: intent-to-treat with censoring at the start of ART or off-protocol censoring where data was censored for subjects who have a primary outcome or a protocol violation. Linear mixed-effects models were used to analyze censored participant data including random effects models to account for repeated measures correlation at the patient level for these analyses. Only individual participants who have measures out to 36 weeks after data censoring were included in the analysis. The mean slopes were reported over 52 weeks for Control and Treatment groups. The differences between mean slopes for Control versus Treatment groups are reported with the 95% confidence interval being given for each as well as the p-value. An alpha level of 0.05 was considered significant.

CD8 T lymphocyte counts (cells/μL), CD4:CD8 ratio and serum chemistries were subject to a per-protocol analysis as described above, censoring for those off-protocol. Serum chemistries measured include: albumin (g/L); alanine aminotransferase (ALT, international units/L – IU/L); alkaline phosphatase (IU/L); amylase (IU/L); aspartate aminotransferase (AST, IU/L); bilirubin (total, μmol/L); blood glucose, random (mmol/L); blood urea nitrogen (BUN, mmol/L); C-reactive protein (mg/L); creatinine (mmol/L); and total protein (g/L). Linear mixed-effects models were used to analyze censored data using random effects models to account for repeated measures correlation at the patient level for these analyses. The mean slopes over 52 weeks were calculated for Control and Treatment groups. The differences between mean slopes for Control versus Treatment groups are reported with the 95% confidence interval being given for each as well as the p-value. An alpha level of 0.05 was considered significant.

For serum chemistries, the medians and arithmetic means (with standard deviations) were reported for each study visit (Control or Treatment groups; 0 Weeks, 12 Weeks, 24 Weeks, 36 Weeks, 48 Weeks, 60 Weeks, 72 Weeks, 84 Weeks or 96 Weeks). The % frequency high or low was reported based on the Eastern Ontario Regional Laboratory Association (EORLA) normal reference range. The means from each group were subjected to ANOVA and multiple comparison analysis with an alpha level of 0.05 considered significant.

The EuroQoL group three-level survey was used to survey participants for five parameters associated with Quality of Life (QOL: EQ-5 dimension-3 level survey): activity, anxiety, mobility, pain and self-care. Each participant rated their QOL with regards to the parameter as: 1) no problem; 2) some problems; or 3) extreme problems. All QOL measurements were taken at each study visit (every 12 weeks) until 96 weeks or participant discontinuation. For simplicity, the categories of 2) some problems and 3) extreme problems were combined. Data was reported as the percentage of persons with no problems for either Control or Treatment groups at each study visit (0 weeks, 12 weeks, 24 weeks, 36 weeks, 48 weeks, 60 weeks, 72 weeks, 84 weeks or 96 weeks). No statistics were calculated, as these are a change in proportion rather than individual changes over time.
