# Supplementary material for: A randomized control trial of high-dose micronutrient-antioxidant supplementation in healthy persons with untreated HIV infection
Source: PLoS One. 2022 Jul 14;17(7):e0270590. doi: 10.1371/journal.pone.0270590 (PMC9282469; doi:10.1371/journal.pone.0270590)
Supplement: Attachment — Submitted filename: Plos one review.docx [file pone.0270590.s027.docx]

General:

This manuscript summarized the results from a randomized, double-blind, placebo-controlled multicenter clinical trial in Canada to assess if high doses of micronutrient plus antioxidant supplementation might provide significant beneficial effects on the HIV disease progression in asymptomatic ART-naïve HIV patients compared to similar patients receiving RDA supplementation (control). Based primarily on time-to first detection of CD4 <350 cells/ul, or ART initiation or AIDS-defined illness, no significant difference in disease progression between these two groups were found. The study represents a large volume of work accumulated for over 2 years, and the trial protocol and analyses were considered rather rigorous and thus the data seem to be of high quality, and the findings were consistent with the majority opinions/reviews in the field. The treatment group also received high dose antioxidant supplementation which renders this study more unique and the results worthy noticing. Nevertheless, the impact on key indices of oxidative stress and antioxidant capacity are missing but desirable. Other issues are listed as following.

Specifics:

1. It is believed that the inclusion of multiple antioxidants and minerals in the treatment group (in addition to high doses of multivitamins) was designed to address the compelling hypothesis that oxidative stress plays an important role contributing to the pathogenesis of HIV/AIDS. However, key blood indices of oxidative stress (e.g., F2-isoprostane, 4HNE-His, TBARS etc., and/or antioxidant decreases (e.g., GSH, oxidant scavenging capacity etc.) are missing which were obtainable from the patients’ plasma/blood samples. It would be highly relevant to the theme of the study if giving RDA levels of multivitamins alone would be sufficient to improve the antioxidant/oxidative indices from the baseline comparing to giving high doses of multivitamins and antioxidants.
2. The rate of CD4 T cell decline appeared to be substantially slower in the treatment (-42.7 cells/ul vs -79.7 cells/ul per 52 weeks for the control); yet statistical significance was not reached due to the stringent protocol sensitivity analysis. In a published work, it was suggested that the beneficial effects of micronutrient supplementation could only be solidified if the HIV infection was at an earlier stage. It is unclear from the study described whether such a criterion (of early HIV infection) was taken into consideration for the patient selection. Or if the patients might come from different stages of HIV infection and thus contribute to a higher degree of variability for the CD 4 T cell decline.
